# Characterization of patterns and variability in the dynamics of outdoor aquatic mesocosms: exploring the capabilities and challenges in data supporting aquatic system models

**DOI:** 10.1007/s10646-023-02685-0

**Published:** 2023-07-25

**Authors:** Ann-Kathrin Loerracher, Jürgen Schmidt, Peter Ebke, Amelie Schmolke, Farah Abi-Akar, Nika Galic, Roman Ashauer

**Affiliations:** 1 Mesocosm GmbH, Homberg (Ohm), Hesse, Germany; 2 Waterborne Environmental, Leesburg, VA USA; 3 Syngenta Crop Protection AG, Basel, Switzerland; 4 Department of Environment and Geography, University of York, York, UK

**Keywords:** Aquatic mesocosm, Mechanistic effect models, Ecosystem, Risk assessment, Model parametrization, Historical control data

## Abstract

Aquatic mesocosms are complex test systems used within regulatory risk assessment of plant protection products. These model ecosystems allow researchers to capture interactions of multiple species under realistic environmental conditions. They enable assessment of direct and indirect effects of stressors at all trophic levels (i.e., from primary producers to secondary consumers) and impacts on ecosystem functions. Due to the limited ability to test the multitude of potential exposure scenarios, cross-linking aquatic mesocosm studies with virtual mesocosms, i.e., aquatic system models (ASMs), can serve to meet the demand for more environmental realism and ecological relevance in risk assessment. In this study, full control data sets from seven aquatic mesocosm studies conducted at a single test facility under GLP were analysed graphically and using descriptive statistics. Thereby, not only a comprehensive data base but also an insight into the species present, their dynamics over time, and variability in unchallenged mesocosms was observed. While consistency in dynamics could be discerned for physical and chemical parameters, variability was evident for several biological endpoints. This variability points to amplification of small differences over time as well as to stochastic processes. The outline of existing gaps and uncertainties in data leads to the estimation of what can be expected to be captured and predicted by ASMs.

## Introduction

Outdoor aquatic mesocosms, artificially set up model ecosystems, are regularly used as higher-tier test systems in environmental risk assessment and authorization of plant protection products (PPPs; Aquatic Guidance Document EFSA PPR panel 2013; Caquet 2013). They are constructed using environmental samples (e.g., sediment and water) or enclose parts of surface water bodies, thereby maintaining key structures of natural ecosystems, such as interspecies competition for resources and nutrient cycling (EFSA PPR panel 2013). With their high complexity, containing an assemblage of interacting key taxa from multiple trophic levels (e.g., macrophytes, phytoplankton, periphyton, zooplankton, and macroinvertebrates), aquatic mesocosms allow for assessment of potential impacts – direct or indirect—that stressors such as PPPs may have on environmental aquatic populations and communities, e.g., in edge-of-field surface waters (Caquet et al. 1996). Moreover, they allow for replication, determination the fate of the test compound, understanding and assessing of delayed effects, multispecies interactions, and ecological recovery options under environmentally realistic conditions (Crossland and La Point 1992, Peither et al. 1996, Gergs et al. 2016).

In the context of authorization of PPPs, aquatic mesocosm studies have found application in environmental risk assessment to derive ‘regulatory acceptable concentrations’ in Europe for more than 25 years (Caquet et al. 2000, Caquet 2002). To date, several guidance and advisory documents have been published (e.g., Campbell et al. 1999, Brock et al. 2000a, Brock et al. 2000b, Brock et al. 2015, Giddings et al. 2002, EFSA PPR panel 2006, EFSA PPR panel 2013, OECD 2006, De Jong et al. 2008) that have led to recommendations for a gradual harmonization in system set-up, validity criteria, experimental design, statistical analysis and interpretation, which may have increased the reliability and statistical power of mesocosm studies. Current requirements that aquatic mesocosm studies must meet in order to be utilized for PPP risk assessment include the presence of a representative aquatic community comprising key taxa of different trophic levels and a minimum number of potentially sensitive taxa (EFSA PPR panel 2013). Depending on the purpose of the study, the design of the test system can be optimized, e.g., by introducing potentially sensitive/vulnerable species (e.g., crustaceans and macrophytes), shading, or the installation of nets to repel birds.

Owing to the complex interplay of many factors and processes, exposure scenarios of PPPs experienced by environmental aquatic communities in the field are highly variable. Exposure patterns originate from the interplay of factors such as successive pulsed applications (FOCUS 2001), distinct ways of entry (e.g., rain-event-driven runoff, spray drift and drainage (Reichenberger et al. 2007), sorption and desorption to sediment and soil particles (Margoum et al. 2006), and different degradation kinetics depending on environmental conditions (Lartiges and Garrigues 1995, Racke 2003). However, high requirements with respect to infrastructure, manpower and resources for system management, sampling and data analysis constrain the ability to experimentally test the multitude of potential exposure scenarios. Typically, effects of static exposures after single or multiple applications, replicated at different concentrations are investigated in mesocosm studies. This leads to a specific uncertainty, as it remains unknown how the aquatic community would have responded to different time-variable exposure scenarios (Brock et al. 2015). Considering the above, and to meet the demand for more environmental realism and relevance (SCHER 2013), risk assessment of PPPs may benefit from the application of mechanistic effect models, especially aquatic system models (ASMs), to complement and extend data from mesocosm studies.

ASMs are simulation models that represent multiple species across trophic levels and can simulate effects of chemical stressors including their cascading effects through the food web (Park et al. 2008, Kattwinkel et al. 2016, Strauss et al. 2017, Bartell et al. 2018). By capturing and predicting multi-species dynamics in aquatic mesocosms, i.e., developing and applying ‘virtual mesocosms,’ ASMs can be valuable tools for PPP risk assessment. ASMs enable extrapolation of effects across different exposure scenarios and may address the question of how a system’s community would have responded to a different temporal exposure profile. Moreover, ASMs could assist extrapolation of effects across levels of biological organization, e.g., organism-level effects to population or community levels (O’Neill et al.^1982^, Traas et al. 2004, Bartell et al. 2013, Kattwinkel et al. 2016, Strauss et al. 2017, Galic et al. 2019). In doing so, they could help (I) to make better use of empirical data, (II) to identify missing pieces in empirical data sets, and (III) to enable more targeted experimental and sampling designs in mesocosm studies. However, with respect to their applicability for regulatory risk assessment of PPPs, it first needs to be demonstrated that ASMs have the capability to capture expected ecological patterns and population dynamics found in the empirical systems (e.g., aquatic mesocosms) - and this, ideally, under natural conditions as well as under any treatment of interest. This is accomplished by model calibration to available empirical data, i.e., an adjustment of the key model parameters until the model output sufficiently resembles patterns observed in the empirical data. This process is followed by validation to assess reliability and robustness of a model to predict system dynamics observed in independent empirical data that have not been used in any prior part of the model development (Rykiel 1996, EFSA 2014 PPR panel 2014).

Among the first steps in calibration and validation of a model is the identification of the main properties and drivers of dynamics and patterns observed in the empirical data. This is critical since success of calibration and validation depends on understanding and quality of the underlying empirical data (Bennett et al. 2012, Schmolke et al. 2020). The more consistency that can be identified in the empirical data, the easier it will be to evaluate how well a model can represent and predict dynamics and patterns in the system of interest. However, dynamics and patterns in aquatic ecosystems are shaped by a complex interplay of various factors that lead to a ‘normal background variability’ which pose a significant challenge for modeling and model evaluation. In some cases, ASMs might be able to capture and explain this variability and thus can make better use of empirical data. In other cases, models may be less successful at capturing observed variability, but their use will still be advantageous as their application could help to identify missing pieces in empirical data sets. As a result, more targeted experimental and sampling designs could be implemented in mesocosm studies, which will further improve the development and application of both aquatic mesocosms and ASMs in the future.

Design, calibration, and validation of ASMs will enhance environmental realism and relevance of risk assessment for PPPs. ASMs rely on a profound understanding of aquatic mesocosms themselves, and the consistency and variability in the dynamics and patterns of these systems without chemical treatments. To address the latter, this study offers a detailed insight into the structure, design, and sampling regime of mesocosm studies and highlights differences that result from different requirements for testing different compounds. In addition to the analysis, we provide the full control data sets of untreated mesocosms from seven studies conducted under GLP at the same test facility in Germany.

In the first approach of analysing the mesocosm control data published with this paper, a focus was placed on addressing the following questions:

- What are the similarities and differences in physical and chemical characteristics of mesocosms within and across studies?
- What are the most abundant and present taxa across all studies? How consistent are the properties, e.g., peak timing of abundance and emergence of the most abundant taxa within and across studies?
- How similar or different are dynamics of most relevant taxonomic groups across studies vs. within studies?

In the light of these results, opportunities for mesocosm studies to support ASMs for application in risk assessment are discussed. In this context, possible supplements, and modifications in the existing sampling regime of mesocosm studies are presented.

## Materials and methods

### Aquatic mesocosms

Data from untreated controls in seven aquatic mesocosm studies conducted by MESOCOSM GmbH in 2014 (Control 14a), 2016 (Control 16a), 2018 (Control 18a, 18b, and 18c), and 2019 (Control 19a and 19b) were analyzed in this study (see Table 1). The studies, each conducted with five to six (control) replicates, were developed for effect assessment of PPPs in edge-of-field surface waters. All studies were performed in compliance with Good Laboratory Practice (GLP), followed the latest guidelines for testing of PPPs issued by the Organization for Economic Co-operation and Development (OECD) and the European Food Safety Authority (EFSA) and met the validity criteria (e.g., acceptable MDD for ≥8 potentially sensitive taxonomic groups) specified by these documents (OECD 2006, EFSA PPR panel 2013).

**Table 1:** Study-specific details of the design and preparation of the test systems

	Pond system			Enclosure (mesocosm)				In life-phase	
Name of data set (Mesocosm GmbH ID)	Date of prepar-ation	Diameter (Volume)	Equilibration time [months]	Diameter (Height)	Water level	Water volume	No. of control enclo-sure	Study start	Study duration [days]
Control 14a (1478–04)	Apr. 2013	9.39 m (~76000 L)	12	1.43 m (1.50 m)	110 cm ±20%	~1700 L	6	12th May 2014	96
Control 16a (1478–10)	Nov. 2014	9.39 m (~76000 L)	18	1.43 m (1.50 m)	110 cm ± 20%	~1700 L	6	17th June 2016	98
Control 18a (1404–48)	Oct. 2015	9.39 m (~76000 L)	30	1.43 m (1.50 m)	100 cm ± 20%	~1600 L	5	7th May 2018	101
Control 18b (1506–02)	Oct. 2016	7.68 m (~55000 L)	19	1.43 m (1.50 m)	110 cm ± 20%	~1700 L	5	4th June 2018	95
Control 18c (1502–02)	Nov. 2016	7.68 m (~55000 L)	17	1.30 m (1.50 m)	110 cm ± 15%	~1460 L	5	22th May 2018	95
Control 19a (1510–02)	Apr. 2018	9.39 m (~76000 L)	12	1.43 m (1.50 m)	110 cm ± 15%	~1700 L	5	13th May 2019	95
Control 19b (1404–50)	Nov. 2018	9.39 m (~76000 L)	6	1.43 m (1.50 m)	110 cm ± 10%	1700 L	6	04th June 2019	95

The dates listed as study start refer to the first day of sampling

#### Location and preparation of the test systems

All studies were conducted in enclosure systems, i.e., series of stainless-steel enclosures (mesocosms) placed in large artificial ponds, located at the test facility of MESOCOSM GmbH in Homberg (Ohm), Germany (50°45’10.8"N 9°01’56.3"E). The ponds, consisting of a concrete base and a circular wall of steel coated with glass-enamel, were lowered approximately 1 m into the ground to reduce temperature fluctuations. Each pond was initially filled with a basal clay layer of 5–15 cm, on which a sediment layer of about 10 cm was added on top. Both clay and sediment were sourced from the test facility, i.e., clay from a natural deposit and sediment from the upper 20 cm horizon of a lake with no known history of contamination. Washed and sieved sand was added to adjust the total organic content (TOC) of the sediment layer to 1–3%. The ponds were filled with water, which was also sourced from a large semi-natural pond at the test facility and mixed with rainwater. This procedure allowed for transfer of macroinvertebrates, zooplankton and phytoplankton from local natural ecosystems into the artificial ponds. Macrophytes, e.g., *Myriophyllum spicatum*, were introduced from a permanent outdoor culture of MESOCOSM GmbH.

Following these initial preparation steps, the ponds were allowed to equilibrate to allow development of a community representative of lentic surface water bodies (as demanded by current guidelines; OECD 2006, EFSA PPR panel 2013). The equilibration period lasted for at least 6 months (for study-specific details see Table 1) and was driven by the study objective and the compound tested. Shorter equilibration periods are favorable when testing effects of herbicides on primary producers, e.g., algae, which typically tend to be highly abundant during the first months after set-up of the ponds. Longer equilibration times (>1a) are generally chosen for testing effects of insecticides, as, e.g., aquatic arthropods that could potentially be sensitive and typically have longer development periods, are then present in higher abundances.

At least 5 weeks prior to the start of the study, enclosures were pressed into the clay layer, isolating sediment, water, and organisms from the rest of the pond. Thereafter, all enclosures were harmonized with respect to density of macrophytes, which involved harvesting of *Chara globularis* and planting of e.g., *Myriophyllum spicatum*.

#### Initial sediment and water characterization

In order to characterize the initial conditions and to exclude contamination prior to study initiation, sediment and water samples were analyzed (non-GLP) for metals (e.g., Cd, Cr, Cu, Fe, Hg, Mn, Ni, Pb, Zn), 6 polychlorinated biphenyls (PCB 28, PCB 52, PCB 101, PCB 153, PCB 138, and PCB 180), 27 pesticides (e.g., aldrin, dieldrin, and heptachlor), grain size fractions, dissolved organic carbon (DOC), total organic carbon (TOC), and other chemical parameters such as Na, K, S, and Cl.

All samples contained non-quantifiable concentrations of all the polychlorinated biphenyls and pesticides analyzed. Heavy metal concentrations measured in the sediments met quality targets of Level I according to German LAWA 1998.

#### Pre-study management

Pre-sampling of all biological endpoints was conducted to assess conditions of individual enclosures, e.g., abundances of potentially sensitive taxa. Based on the results, it was decided which enclosure to include in the study to achieve the best comparability, and whether additional interventions were necessary. Interventions included, e.g., introduction of relevant and/or potentially vulnerable species, e.g., *Gammarus pulex* and *Asellus aquaticus*. For study-specific details on species introduced, see Table SI 1. If needed, macrophytes managed to reach a degree of coverage of ≤25 % at the start of the test.

#### Study-in-life management

When management actions were implemented during the in-life phase of a study (i.e., after first sampling occasion), these were driven by specific properties of the test compound or special requirements of organisms in terms of water quality. For instance, to lower the rate of degradation of a test compound that degraded faster at alkaline than at neutral or acidic pH, which occurred in studies 18b and 18c, a tent was temporally installed to reduce photosynthetic active activity and thus mitigate the rise in pH associated with photosynthesis. Other management actions included artificial aeration to prevent oxygen depletion due to relatively high temperatures (study 18c), and installation of pumps, which was favorable when, e.g., the freshwater amphipod *Gammarus* was introduced into the test system (study 19a; for study specific details see Table SI 2).

#### Sampling regime and investigated endpoints

The studies were conducted following similar procedures, which allowed for comparison among the seven studies. All sampling methodologies were standardized. Differences were thus mainly limited to sampling regime (i.e., sampling dates and intervals), which were carefully selected to best capture both direct and indirect effects related to the specific properties of the respective test compounds (e.g., expected short-term effects and degradation kinetics). However, as sampling events, destructive or non-destructive, pose stress on the test system, the number of sampling events taken within a mesocosm study should be as few as possible but as frequent as necessary to capture potentially occurring effects and/or recovery. For instance, for zooplankton, short sampling intervals are favorable during the first one to two weeks after application, since the fast recovery potential of r-strategists could prevent effects being observed. Thereafter, weekly, or biweekly sampling intervals are often sufficient. For macroinvertebrates weekly or biweekly sampling is generally sufficient.

Depending on the compound tested in the specific study, counting and taxonomical identification of phytoplankton or periphyton taxa was performed. Not every sample taken was analysed thereafter.

**Fig. 1:**
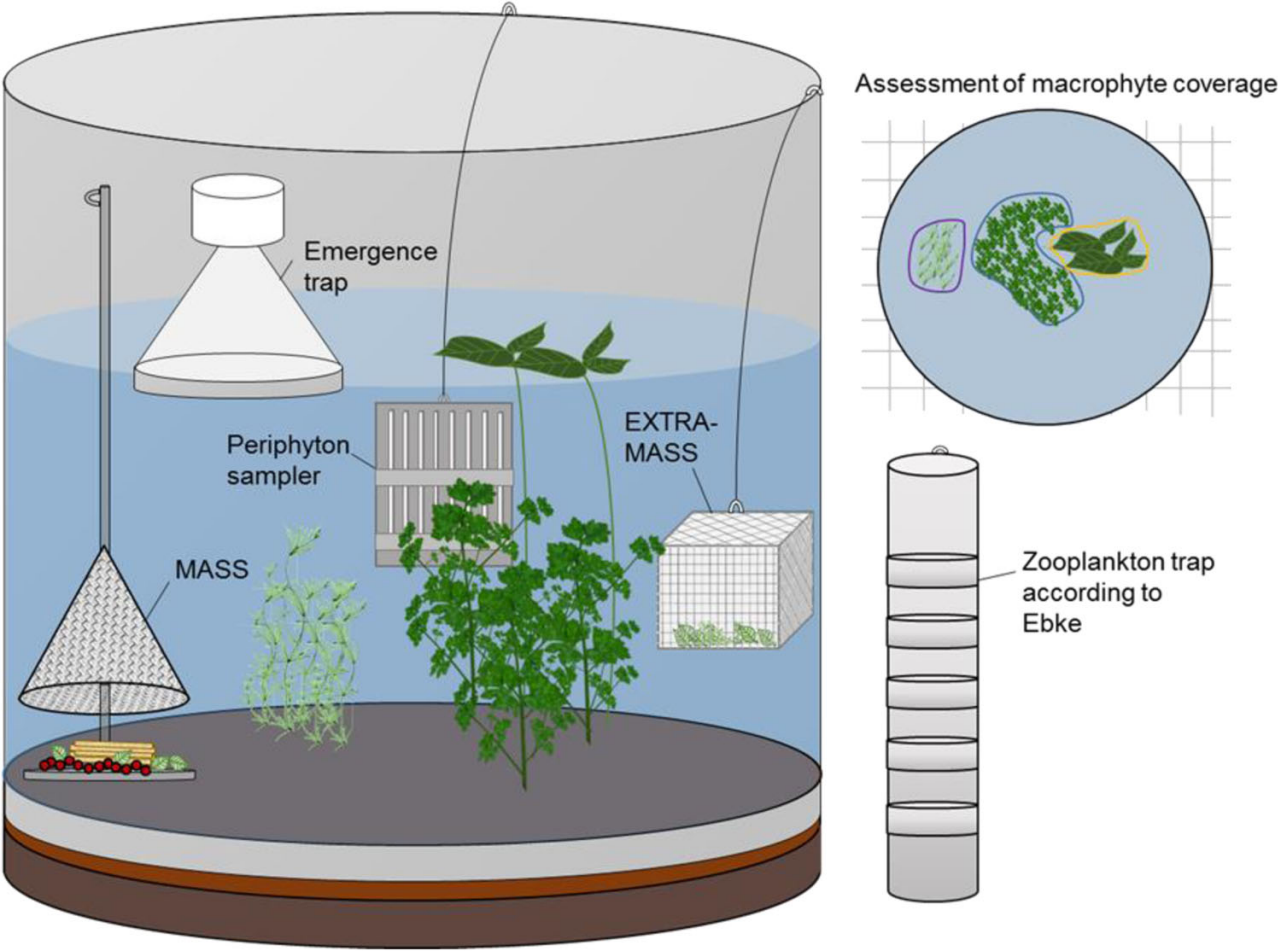
Scheme of mesocosm setup and different types of sampling devices. MASS macroinvertebrate artificial substrate sampler

The different sampling devices were developed mainly in-house and specifically designed to capture potentially sensitive species in high abundance. Some sampling devices (e.g., macroinvertebrate artificial samplers, see Fig. 1) were designed to increase abundance of potentially relevant taxa in samples relative to the environment thereby allowing for statistically reliable calculation and evaluation of data (e.g., according to Brock et al. 2015) and meet validity criteria set by OECD and EFSA guidance documents (OECD 2006, EFSA PPR panel 2013).

##### Weather conditions

Hourly records of air temperature at 2 m height [°C], air pressure [hPa], relative humidity [%], precipitation [mm], wind velocity and wind speed at 10 m above the ground [m/s] during the 4 study years were obtained from the German Weather Service (DWD) station situated approximately 800 m from the test facility in Neu-Ulrichstein. Global radiation data [W/m^2^] were obtained from surrounding weather stations within a distance of 35 km and 70 km [SI Weather-2013-2019_MesocosmGmbH-ID-1404-54).

##### Physicochemical water parameters

Temperature [°C], pH, electrical conductivity [µS/cm], and dissolved oxygen [mg/L] were measured at ~70 cm below the water surface using a WTW multiparameter instrument (WTW, Weilheim, Germany). For chemical analysis, three depth-integrated water samples were taken from each enclosure and combined into a final composite sample, which was sieved (mesh size: 60–70 µm) prior to photometrical analysis using WTW cube test kits for nitrate (WTW 14556), ammonium (WTW 14739), phosphate (P6/25) and water hardness (WTW 00961). The limit of quantification (LOQ) for nitrate concentrations was 0.4 mg/L, for ammonium 0.01 mg/L and for phosphate 0.2 mg/L. Values measured below the LOQ were assumed to be half the LOQ for the calculation of arithmetic means across enclosures.

##### Zooplankton

Zooplankton were collected using a depth-integrated sampler (‘Ebke’s zooplankton trap’, a tube system with closable windows along the longitudinal side; for details, see Fig. 1). The sampler was immersed into the water column with open windows and allowed to remain in situ for at least 5 min. Organisms were collected in a zooplankton sieve (mesh size: 60–70 µm), rinsed with tap water and fixed by adding ethanol to a final concentration of 70–80%. Preserved zooplankton organisms were taxonomically identified and enumerated using a stereomicroscope. Taking into account the exact water volume sampled (dependent on the water level in the respective enclosure), data were expressed as individuals per litre.

##### Macroinvertebrates

Abundance of macroinvertebrates was assessed by three different sampling methods: (I) Sweep netting (three pulls, aperture: 21 × 21 cm, mesh size: 450 µm; estimated water volume sampled: ~170 L), which was used for collecting pelagic or drifting macroinvertebrates. (II) Macroinvertebrate artificial substrate sampler (MASS), open stainless-steel systems (diameter: 20 cm) containing clay beads and decomposed organic material (e.g., leaves of *Populus*), which were placed onto the sediment, and were allowed for sampling of benthic macroinvertebrates. (III) EXTRA-MASS, wire baskets containing macrophytes, installed ~50 cm beneath the water surface, designed for sampling of semi-pelagic macroinvertebrates. One MASS and one EXTRA-MASS were placed in each enclosure and remained in situ for at least 5 days to allow (re)colonization. To prevent organisms from escaping on sampling occasions, EXTRA-MASS samplers were retrieved using a net (mesh size: 450 µm). The macroinvertebrates were taxonomically identified to the lowest practical taxonomic level, counted, and subsequently retransferred into their respective enclosure. Abundance of macroinvertebrates was expressed as individuals per sample, and represent the cumulative number of macroinvertebrates collected from a single mesocosm using netting, MASS and EXTRA-MASS.

##### Emerging insects

Numbers of emerging were assessed using emergence traps remaining in situ over 7-day periods. The emergence traps were constructed of stainless steel carrying an apical trapping device filled with tap water and a small quantity of a surfactant to prevent insects from escaping (see. Fig. 1) . The size and number of traps were adjusted to not cover more than 25% of the mesocosms’ water surface (for study specific details see Table SI 3). All insects captured were fixed with ethanol (70–80%). Preserved individuals were taxonomically identified and enumerated using a stereomicroscope. Data were expressed as individuals per sample. When two traps were used per enclosure, samples from both traps were combined into one. The given values represent the cumulative sum of insects emerged over 7-day periods.

##### Phytoplankton

Phytoplankton (in raw data expressed as µg chlorophyll-*a* per L and/or cells per ml) was sampled using three depth-integrated water samples taken with a stainless-steel tube from the first 80 cm of the water column. The subsamples were combined into a final composite sample and sieved (mesh size: 2 mm) to remove plant debris. The phytoplankton composition was quantitatively and qualitatively analysed using two methods: Determination of the wavelength-specific activity of photosynthetic active pigments (e.g., chlorophylls, fucoxanthin, and phyoerithrin) of living algae using delayed fluorescence (DF) spectrometry (Gerhardt and Bodemer 1998). This determined the concentration of chlorophyll- *a* and the respective fractions of green algae (‘greens’: Chlorophyceae, Euglenophyceae and Conjugatophyceae), blue-green algae (‘blue-greens’: Cyanoprokaryota), ‘diatoms’ (Bacillariophyceae, Chrysophyceae, Dinophyta and Xyanthophyceae), and cryptophytes as described in Gerhardt & Bodemer (1998). This classification may differ from taxonomic classification (for details see Gerhardt & Bodemer 1998, Gerhardt et al. 2005). In studies 18b and 19b, a sample was preserved with Lugol’s solution. Phytoplankton species composition was analysed by taxonomical identification and enumerated using an inverted microscope (magnification: x400), a counting grid and a tubular plankton chamber.

##### Periphyton

Periphyton (in raw data expressed as µg chlorophyll-*a* per cm^2^ and/or cells per cm^2^) was sampled from glass slides (area of approximately 185.5 cm^2^) that served as artificial substrate and were pre-exposed at least five weeks before the start of the study. The slides were positioned vertically in a retainer at 40–60 cm beneath the water surface. The position was kept the same in all mesocosms within one study. On each sampling date, one slide per mesocosm was taken for analysis. Periphyton was scraped off with a razor blade, rinsed into a light proof bottle to dark-adapt algae and filled up with tap water to a final volume of 350 ml. The cleaned slides were re-introduced into their original enclosure but not re-analysed. Content of chlorophyll-*a* and the respective fraction of green algae, blue-green algae, diatoms and cryptophytes (Gerhardt and Bodemer 1998) were analysed using DF spectrometry as described for phytoplankton. In study 19b, algae suspension was additionally preserved using Lugol’s solution and taxonomically identified and enumerated.

##### Macrophytes

Species composition and coverage of macrophytes were visually assessed (vertical projection) and mapped to calculate % coverage of the sediment surface area per species.

### Data analysis

#### Data preparation

Data from seven mesocosm studies (for details see Table 1) were collected and combined for statistical and graphical analysis. Where abundance was recorded by life stage (e.g., larvae, pupae, instar), all were summed and presented as total number for a given taxon. Data were analysed using R software version 4.0.3 (R Core Team 2021) and package ggplot2 was used for graphing (Wickham 2016). All data analysed during the current study are available within the supplementary information.

#### Presence, mean abundance, time-lines, emergences

Within each study, all mesocosm replicates (5-6 per study) were sampled on all sampling dates. In this analysis, one sample was defined as one mesocosm sampling-date (i.e., one replicate enclosure on a single observation date). The sample data were analysed by taxon and study. For each macroinvertebrate and zooplankton taxon, the number of samples with greater than zero individuals (macroinvertebrates) or individuals/L (zooplankton) were counted in each study. This number was divided by the total number of samples to generate a percentage of samples with presence, per study and taxon. Additionally, the mean abundance (arithmetic mean of the number of individuals or individuals/L per sample), was calculated for each study and taxon. Some taxa were only present in a subset of studies. Presence and mean abundance were graphed by taxon (Figs. 3 and 5).

**Fig. 2:**
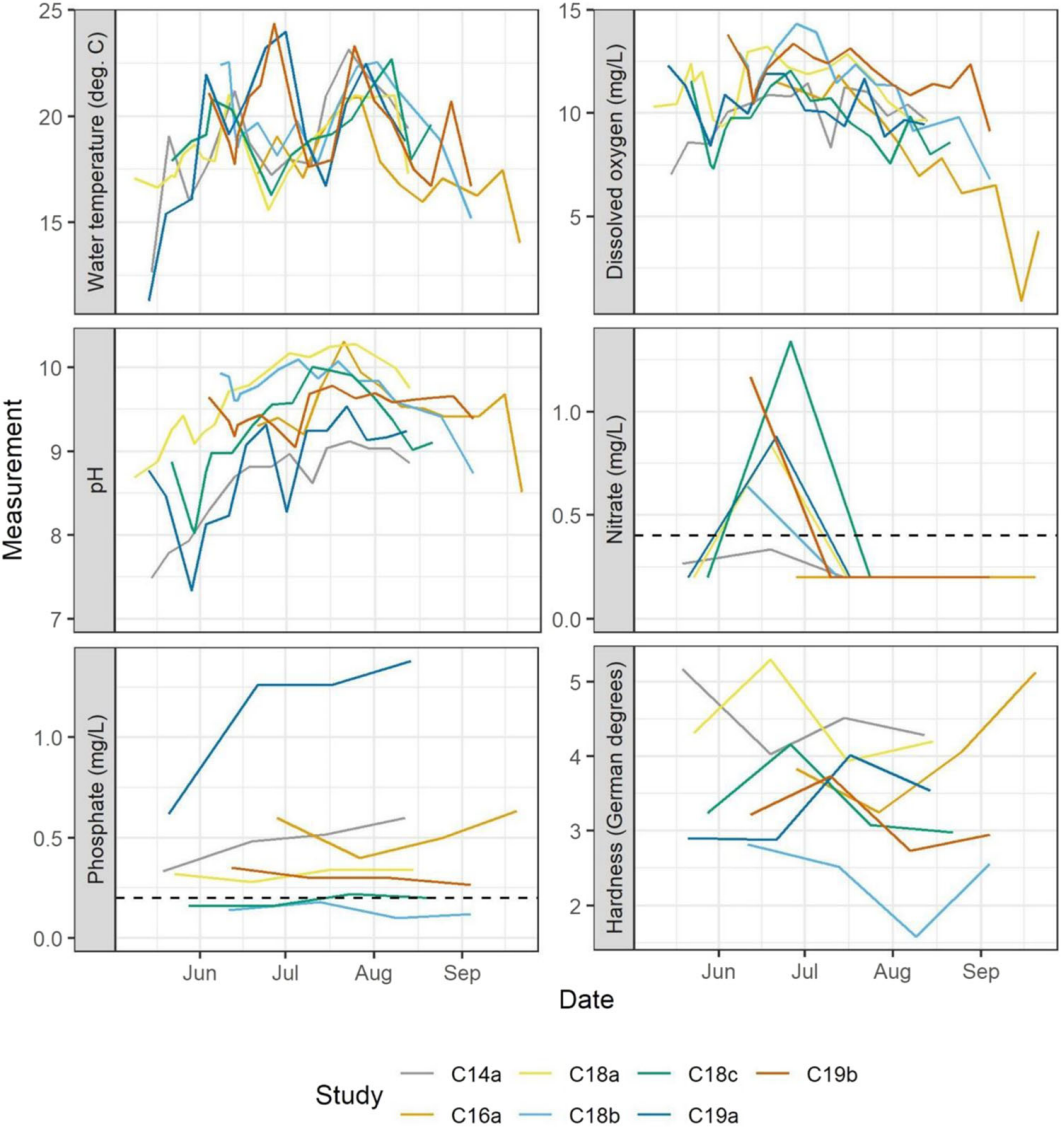
Mean values of physical and chemical water characteristics across control mesocosms in each study are plotted by date. Values below the limit of quantification for nitrate and phosphate are graphed at half the limits

Timelines were generated for all biotic classes, as well as the abiotic measurements of water temperature, pH, phosphorus, nitrogen, and hardness (Fig. 2). All studies were superimposed, aligned by calendar date. Macroinvertebrate and zooplankton taxa were limited to those present in at least 90% of samples in at least one study. The dates at which peak abundances occurred were identified per study and taxon. To do so, the arithmetic mean across enclosures was first calculated, such that one mean value was available per date, taxon, and study.

The date at which the maximum of these mean abundances occurred was identified as the peak date. When a maximum occurred on more than one date (relevant for taxa with low abundance), all these dates were identified as peak dates. This enabled a comparison of peak dates across studies (Figs. 4 and 6).

The dominance of different macroinvertebrate and zooplankton taxa was also calculated to inform the relative abundance of different species within replicates. For each unique taxon, the total number of individuals or individuals/L were summed across all dates, within enclosures. This taxon-specific total was divided by the total of al taxa calculated per enclosure and across dates, to define the proportion. An abundance proportion therefore was calculated for each taxon and enclosure.

Data on insect emergence were prepared for inclusion first by aligning taxa reported from the emergence data with taxa reported in the macroinvertebrate samples collected in the water because the later were reported at higher taxonomic levels in some cases (e.g., in case of *Cloeon diperum* (emergence data) and *Cloeon* sp. (macroinvertebrate data) the lower taxonomic level *Cloeon* sp. was used for statistical analysis). Since traps were collected once a week, emergence counts were divided by seven and assigned to the associated seven-day period (Fig. 7). Emergence data are presented in daily interval to match single-day sampling in water.

The Shannon diversity index was calculated for all macroinvertebrate, zooplankton, and emerged insect samples (SI Fig. 1) using the R ‘vegan’ package (Oksanen et al. 2020).

#### Variability among replicates

The variability of abundances among enclosures, or replicates, was examined as an indicator of consistency in the studies’ methods (Fig. 8, Figs. SI 11 and 12). Depending on the study, five or six replicates exist for each macroinvertebrate and zooplankton taxon, within one study and one date. This is defined as one set, and 3198 sets exist among the seven studies.

First, sets with small means (macroinvertebrates <1 individual, or zooplankton <0.5 individuals/L) corresponded to taxa with low presence (many samples without any individuals reported); therefore, they were omitted from this analysis and the number of sets decreased to 1860. Next, each set was tested for normality using the Shapiro-Wilk test (R Core Team 2021). Since 26% of sets were non-normal (*p* < 0.05), all sets were transformed using ln(Ax + 1), where A is a calculated coefficient and x is the measurement. For macroinvertebrates A = 2 was applied and for zooplankton, A = 33.33. This follows (Van den Brink et al. 2000), a method used in statistical analyses of mesocosm data (see Table SI 4 for transformation details). This transformation reduced the percentage of non-normal sets to 14%. Two calculations were performed for each set, using the transformed values. The coefficient of variation (CV), defined as the standard deviation divided by the mean, was calculated. Also, Rosner’s test (generalized extreme Studentized deviate test) was run to test for one potential unusual enclosure in either direction, using *α* < 0.05 (Millard 2013, 2020). Though it does not identify sets with unusual enclosures or sets with generally wider variance, Rosner’s test was chosen because it tests for outliers in both directions, low and high; it can be run on sample sizes of 5 or 6 (Millard 2020); and it is based in the useful consideration of whether one enclosure is significantly different than the mean of the set (USEPA 2006). All calculations and tests were run in R software (R Core Team 2021).

## Results

### Abiotic (physicochemical) parameters

Records of air temperature exhibited pronounced seasonal dynamics with peak values reached in summer (June—August). Highest air temperature was documented in 2019 with an average monthly temperature of 18.45 °C (min: 12.40 °C; max: 28.80 °C) during July. Water temperature, dissolved oxygen, pH profiles exhibited pronounced seasonal patterns following trends in air temperature. Although dissolved oxygen, pH and water temperature fluctuated and peak heights varied among years and studies, their profiles were quite similar, with all parameters reaching peak values in summer (June-August) before gradually declining over the course of the season (see Fig. 2 and Figs SI 3–9). High consistency was documented across the seven studies with respect to the peak timing of nitrate. In all studies, nitrate reached peak concentrations during mid- to late June, which was consistently followed by a sharp decline in July, when nitrate in all studies reached levels below the limit of quantification (<0.4 mg/L). Phosphate, hardness, and conductivity, on the other hand, did not show any identifiable consistency in patterns across the studies but varied in time and magnitude. Phosphate values ranged from ≤0.2 mg/L (limit of quantification) to 1.80 mg/L (Control 19a); hardness ranged from 1.3°dH (Control 18b) to 5.90°dH (Control 16a); and conductivity ranged from 134 µS/cm (Control 18b) to 254 µS/cm (Control 19a). Likewise, for ammonium, no consistency in patterns could be identified across the studies, likely owing to the relatively low concentrations that exceeded the limit of quantification (0.01 mg/L) only on a limited number of sampling dates.

### Zooplankton

**Fig. 3:**
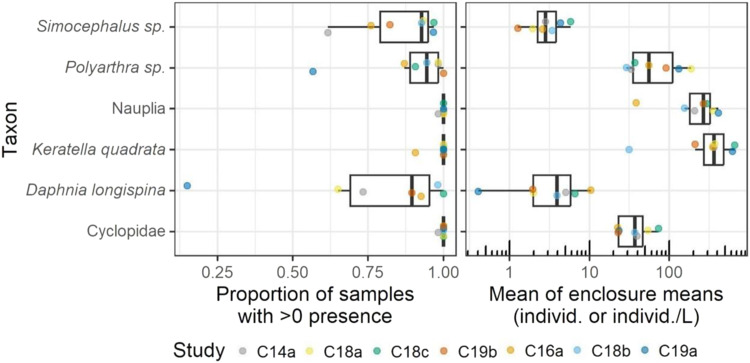
Left: Presence and mean abundance of the most relevant zooplankton taxa across all studies. Right: zooplankton abundances (individuals/L) across all studies. The boxes represent the 25^th^ and 75^th^ percentiles, the whiskers indicate the 25^th^ percentile minus 1.5 times the inter-quartile range and the 75^th^ percentile plus 1.5 time the inter-quartile range, respectively

In total, 54 different taxa were collected using the zooplankton traps. However, here it must be noted that differences existed among the studies in terms of the taxonomic resolution at which single organisms were determined. Among the most abundant zooplankton taxa across all seven studies were nauplia (early larval stage of copepods) and the rotifers *Polyarthra* sp., and *Keratella quadrata*. All these taxa occurred in average densities exceeding 20 individuals/L and were generally more reliably observed across samples than less abundant taxa (see Fig. 3). In the replicates of all studies, except those of study 19a, there was a tendency for diversity to increase as the studies progressed, as indicated by the increase in Shannon index (see Fig. SI 1). Although some temporal variation existed among the control replicates of the seven studies, some species were highly abundant in some months and nearly absent in others. For instance, in all studies except 18b, *Keratella quadrata* were most abundant during June and/or July, after which abundances sharply declined. An opposite trend, with an increase in abundance from June / July was recorded e.g., for Ostracoda. No consistency or obvious seasonal trend was evident in the records of several species, including *Chydorus sphaericus* and *Simocephalus* sp., although abundances of these species considerably fluctuated throughout study (see Fig. SI 2 and Figs SI 3–9).

**Fig. 4:**
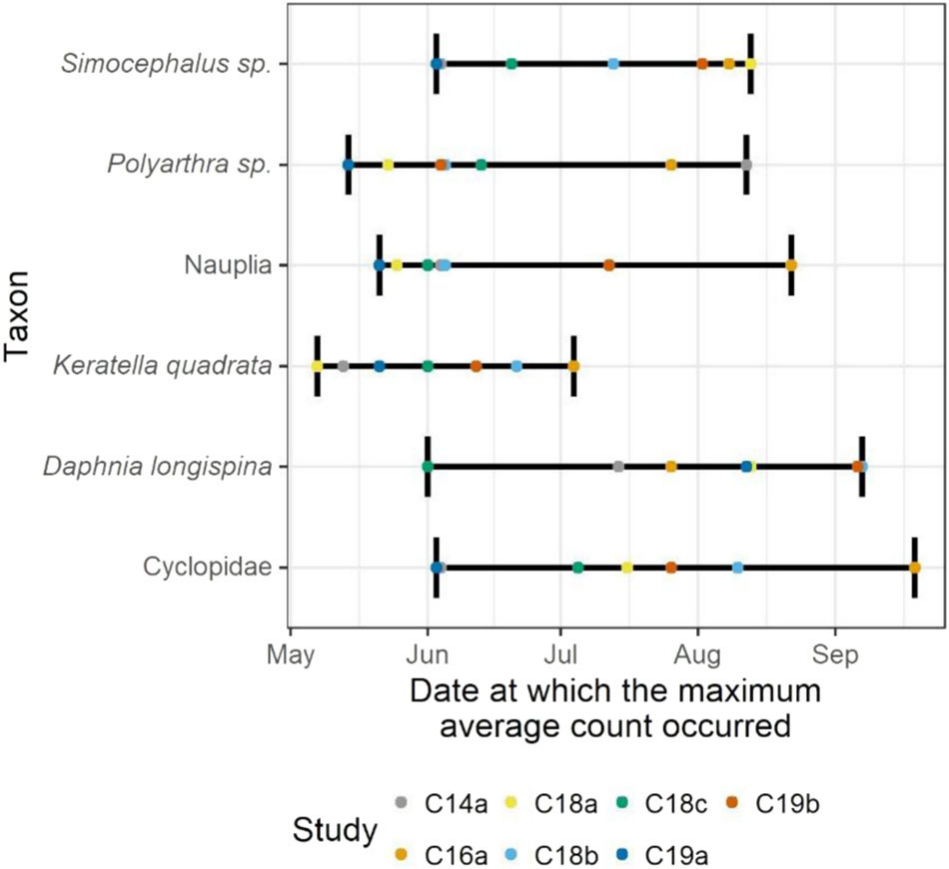
Dates at which the maximum abundance of most relevant zooplankton taxa were reached, across seven studies. Maximums are based on the mean of enclosure measurements

In terms of timing of peak abundance, *Keratella quadrata*, for instance, was characterized by relatively low inter-study variation (i.e., May-July, respectively). This contrasts with the marked variability documented for several other dominant zooplankton taxa, including *Daphnia longispina* and Cyclopidae, where timing of peak abundance varied by up to 4 months (see Fig. 4). Variability across the studies was apparent in terms of the total sum of abundance of zooplankton taxa. For instance, among the seven studies, the peak numbers of individuals/L recorded of *Daphnia longispina* varied from 0 individuals per sample in one enclosure (Control 19a) to 74.0 individuals per sample in one enclosure (Control 16a). Moreover, variability was evident regarding the number of abundance peaks. For instance, the abundance of *Hexarthra* sp. followed a bimodal pattern, with two distinct peaks reached in June and August in study 18b. In contrast, only one peak in abundance was recorded in study 18a for this taxon (see Fig. SI 2).

### Macroinvertebrates

**Fig. 5:**
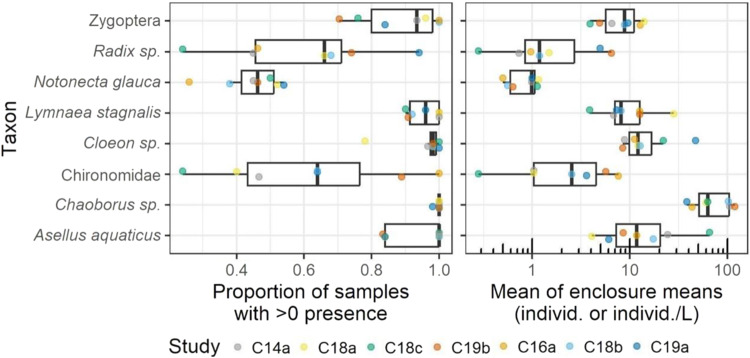
Left: Presence and mean abundance of the most relevant macroinvertebrate taxa across all studies. Right: Average macroinvertebrate counts (individuals/sample) across all studies. The boxes represent the 25^th^ and 75^th^ percentiles, the whiskers indicate the 25^th^ percentile minus 1.5 times the inter-quartile range and the 75^th^ percentile plus 1.5 time the inter-quartile range, respectively

The seven control data sets comprise a total of 11,492 records of macroinvertebrate abundances representing about 52 different taxa, 39 of which were identified to species level. As macroinvertebrates were placed back into their original enclosures after counting, organisms may have been counted multiple times within the same study. Among the seven studies, *Chaoborus* sp*., Asellus aquaticus*, *Cloeon* sp., *Lymnea stagnalis* and Zygoptera were on average the most dominant taxa by abundance, together accounting for a range of 50% (study 19a) to 91% (study 14a) across the different enclosures. These taxa, along with others such as Lymnaeidae <0.5 cm and Chironomidae, were not only found in all studies, but were also present on average in more than 50% of all samples taken (see Fig. 5).

**Fig. 6:**
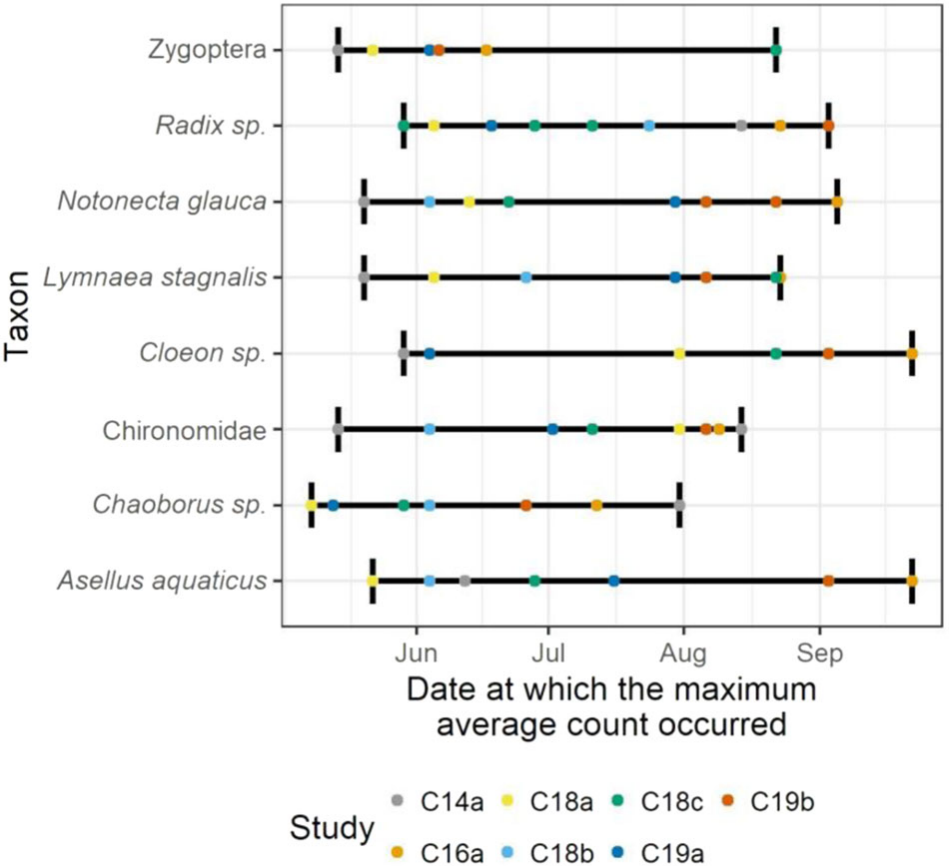
Dates at which the maximum abundance of most relevant macroinvertebrate taxa were reached, across seven studies. Maximums are based on the mean of enclosure measurements

**Fig. 7:**
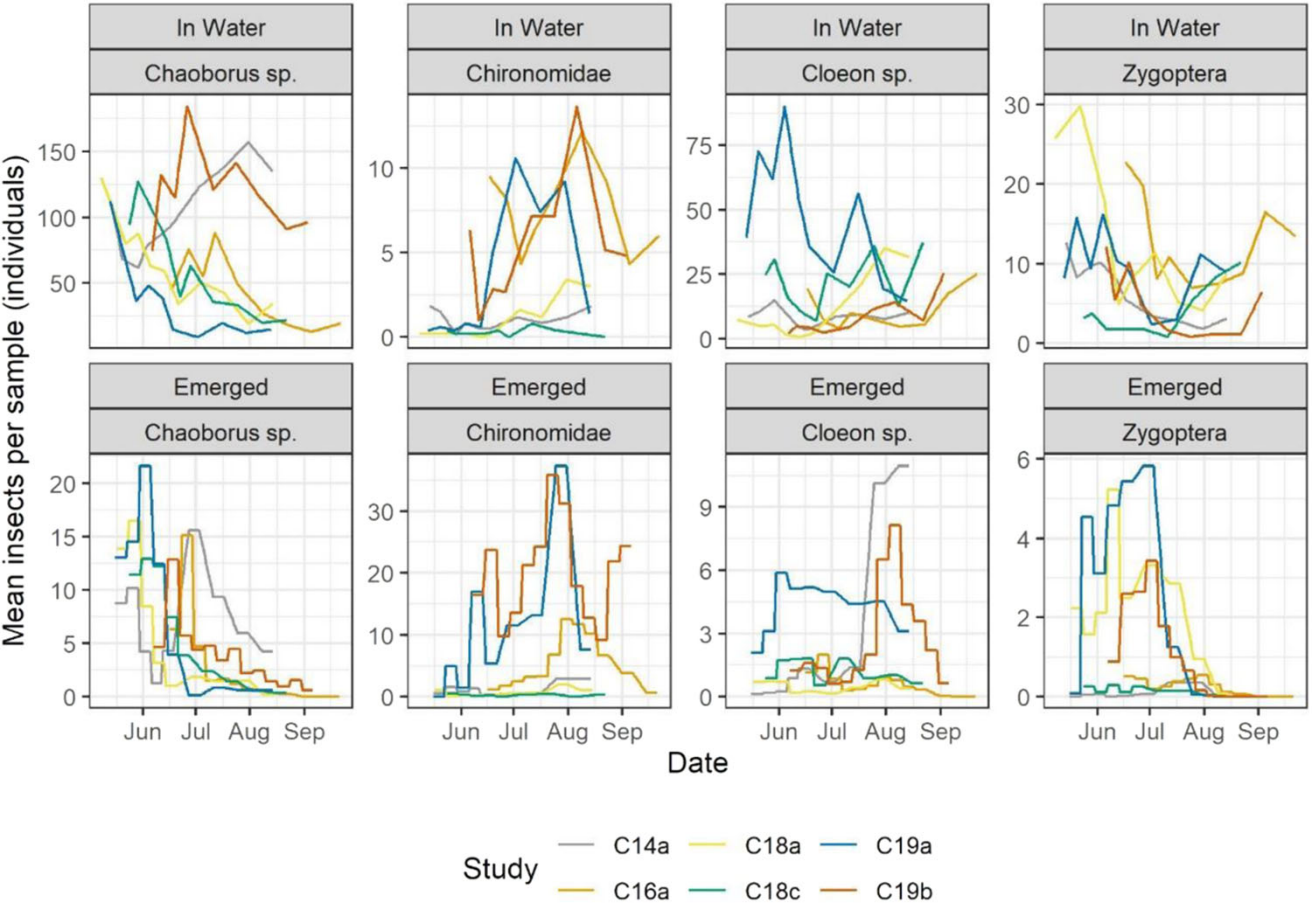
Mean abundances of macroinvertebrates in water samples (top row) and emergences (bottom row) for taxa in the studies with available emergence data. Emergence data are shown as daily numbers (weekly numbers divided by 7). Lines show means of all enclosures by date. Scales vary by plot to allow for lines to be visible

The total number of macroinvertebrates sampled varied considerably among years and studies. The highest numbers of macroinvertebrates were often found in late spring while the lowest numbers were found in August/September. Some species exhibited large differences over the study period: e.g., *Chaoborus* sp. varied from 1 to 167 individuals in one enclosure (study 18a), and *Cloeon* sp. varied from 2 to 109 individuals in another enclosures (study 18c). Some taxa, including *Chaoborus* sp., Chironomidae, *Cloeon* sp. and Zygoptera, occurred with two or more generations within some studies and enclosures, as indicated by increases and peaks in their abundance in water following peaks of emergence (see Fig. 7 and Figs. SI 3–9). However, the number, magnitude, and timing of abundance peaks varied among years and studies, at least in part due to differences in study duration. While, e.g., for *Chaoborus* sp. the timing of peak abundance varied by 84 calendar dates over the 4-year period, the peak timing of other species, such as *Cloeon* sp. and *Asellus aquaticus*, varied by 116 and 123 days, respectively (see. Fig. 6). Variability among studies and taxa was also documented regarding trends in abundance of macroinvertebrates in water and dates of emergence. Often, changes in the number of taxa approximately co-occurred in samples taken in the water and in the emergence traps (see Fig. 7).

### Emerging insects

A total of 45,246 individuals within 13 taxa were collected in the control emergence traps of the seven studies. Some identifications were to a higher taxonomical resolution (species level) compared to their corresponding larvae in the macroinvertebrate samples; to ensure a standardized comparison, they were grouped within the same taxa categories as in the macroinvertebrate data. Comparisons in terms of the total number of individuals identified per macroinvertebrate and emergence samples are not possible due to differences in sample sizes and dimensions. However, while for some taxa, including *Chaoborus* sp. (~150 individuals) and *Cloeon* sp. (~80 individuals) comparable number of individuals were documented in emergence samples and macroinvertebrate samples, for other taxa, including e.g., Chironomidae, considerably higher numbers of individuals were documented in emergence samples than in macroinvertebrate samples (see Fig. 7). Consistency across studies was evident in terms of the timing over which emergences of specific species were observed. For instance, the great majority of Zygoptera emergence occurred before August. Apart from study 14a, a very similar time window was observed for trapping of *Chaoborus* sp. In contrast, Chironomidae were consistently caught over the entire course of the studies, albeit with considerably varying numbers.

Variability across the studies was apparent in terms of abundances of several emerging insect taxa. For instance, for the abundances of Chironomidae, studies 19a and 19b, with peak emergence values of up to 37.3 and 35.8 individuals (means among enclosure samples, divided by 7), respectively, clearly stood out from all other studies in which abundance values of 12.5 individuals were not exceeded. However, direct comparisons must be interpreted with caution, as there were differences between the studies in the percent coverage of the enclosure area covered by the emergence traps (i.e., 7.8–22.3%, for details see Table SI 3).

### Phytoplankton

The results derived from the spectrophotometric determination of chlorophyll-*a* in the control enclosures of the seven studies are presented in Figs. SI 3–9. In all studies, the temporal dynamics of chlorophyll-*a* concentrations of ‘green algae’, ‘blue-green algae’ and ‘diatoms’ was correlated (see Fig. SI 10 and Tab. SI Tabs. 5–7); however, peak heights varied considerably among the different ‘taxonomic groups’. In most control enclosures, except e.g., single control enclosures of studies 18a, 18b and 19b where ‘cryptophytes’ dominated, ‘green algae’ made up highest levels of chlorophyll-*a*, with an overall peak value of 69.8 µg/L (see control data 18c). Total chlorophyll-*a* concentrations (sum across types) fluctuated considerably over the study periods, with values ranging from 0.95 to 106 µg/L (minimum and maximum measured in control 18c), but no consistency in seasonal or annual patterns was evident. Likewise, no seasonal or annual consistency could be detected in the development of the fraction of ‘green algae’, ‘blue-green algae’, ‘diatoms’, and ‘cryptophytes’ (i.e., the composition of the phytoplankton populations).

In studies 18b and 19b, a total of 51 taxa of green algae, 12 taxa of blue-greens, 38 taxa of diatoms, and 4 taxa of cryptophytes were identified by taxonomical evaluation.

### Periphyton

Spectrophotometric measurements of chlorophyll-*a* concentrations in periphyton suspensions and the determination of the respective fractions of ‘green algae’, ‘blue-green algae’, ‘diatoms’ and ‘cryptophytes’ indicated a clear dominance of the ‘green algae’ in all control enclosures except those of study 18c (see Figs. SI 3–9). The ‘cryptophytes’, on the other hand, generally accounted for only a minor portion of the overall chlorophyll-*a* concentration measured during the course of the studies. Likewise, as it was documented for phytoplankton, in the control enclosures of all studies the dynamics of ‘green algae’, ‘blue-green algae’ and ‘diatoms’ were correlated in time (see Fig. SI 10 and Tab. SI Tabs. 5–7). Variability was documented in terms of peak heights among these different ‘taxonomic groups’. Moreover, no general seasonal pattern in the development of the fractions of ‘green algae’, ‘blue-green algae’, ‘diatoms’, and ‘cryptophytes’ nor in the concentration chlorophyll-*a* could be detected.

The 91 taxa of periphyton determined in study 19b *via* counting were assigned among the taxonomical groups of green algae (47 taxa), blue-green algae (11 taxa), cryptophytes (3 taxa) or diatoms (27 taxa). *Anabaena cylindrica* cf. and *Coenochloris* sp. cf., belonging to blue-green and green algae, respectively, were the most dominant taxa in study 19b, with maximum abundance values of 591,890 and 276,498 cells/cm^2^, respectively.

### Macrophytes

Despite comparable management strategies for macrophytes across studies, the number of macrophyte species recorded in the control enclosures ranged from 3 to 6, with *Myriophyllum spicatum* and *Chara globularis* being present in all. These two species were also the most dominant in terms of the fraction of enclosure-area they covered (see Figs SI 3–9). While *Myriophyllum spicatum* clearly dominated in the control enclosures of four studies (Control 16a, 18a, 18b, and 18c), covering up to 61% of the enclosure area, *Chara globularis* dominated in the control enclosures of the remaining three studies (Control 14a, 19a and 19b), albeit with a coverage not exceeding 59% of the enclosure area. *Ceratophyllum demersum* nearly matched coverage with *Chara globularis* in studies 14a and 16a. In all studies, the overall macrophyte coverage increased over the course of the season. *Zannichellia palustris* developed in studies 14a, 18b, and 18c, while filamentous algae developed in all control enclosures except studies 16a and 18a. The occurrence *Zannichellia palustris* and filamentous algae was generally limited in time, and the enclosure area covered by both these plants did not exceed 21% in any of the studies included. No consistency could be found with regard to occurrence and peak timing of *Zannichellia palustris* and filamentous algae across the seven studies.

### Variability among replicates

The variability among enclosures within the same taxon, study, and date (sets of 5 or 6 replicates) was examined using CVs and Rosner’s test. The CVs calculated for these sets were overly influenced by means, as opposed to the intended focus of standard deviations: higher CVs exclusively occurred in sets with smaller means (Fig. SI 11). Rather, Rosner’s test was more relevant, since it successfully identified sets with one unusually high or low enclosure compared to the others, across all means.

**Fig. 8:**
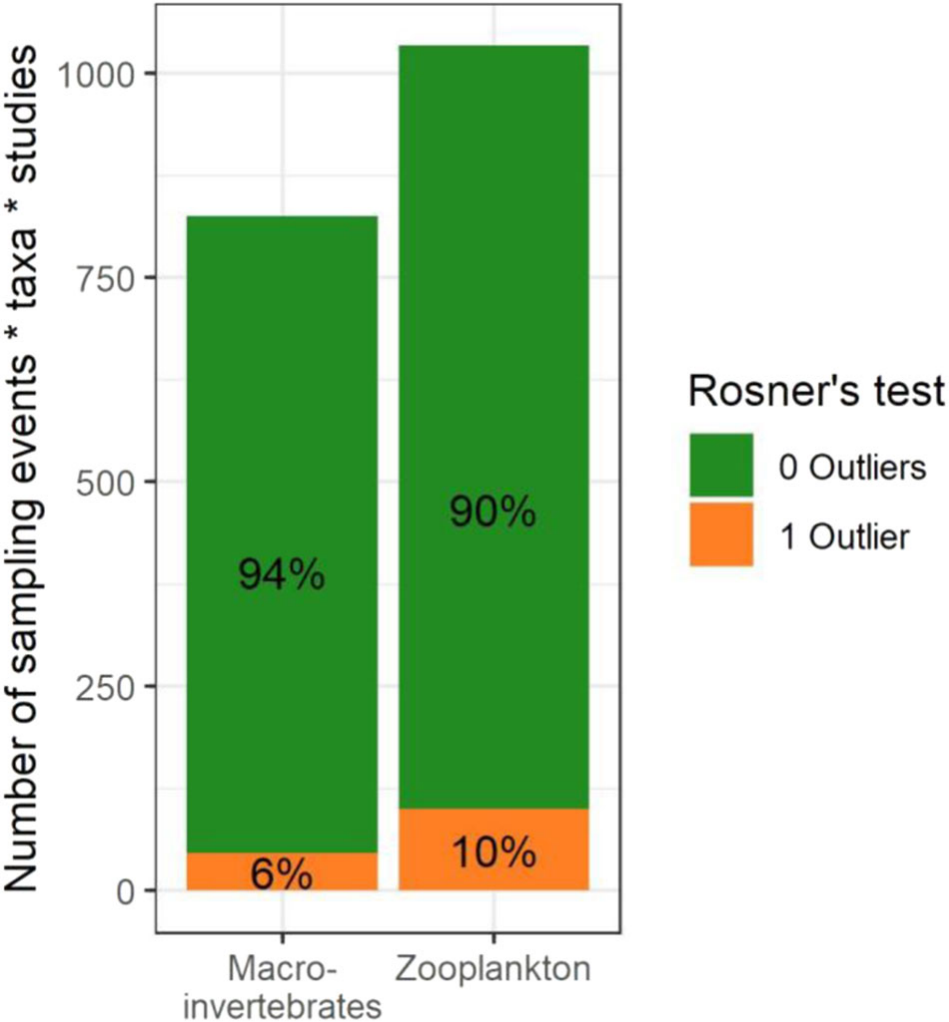
Results of Rosner’s test, as number and percentage of sets with a high or low outlier among all studies

Overall, ~ 8% of sets (143 of 1860) had an enclosure identified as an outlier (Fig. 8). An outlier, as identified by this test and in this current application, is not intended to suggest an incorrect value; rather, it is a point that significantly deviated from the mean of the remaining enclosures (examples are shown in SI Fig. 12). Sets with outliers were somewhat more common among zooplankton than macroinvertebrates, but varied across taxa, studies, and dates. Among the sets with an outlier, 27% were recurrences of one taxon in one study; e.g., an enclosure with a deviant taxon measurement may have remained so for more than one sampling event (as shown in Supplementary Fig. 12).

Proportionally, 72% of the 143 identified outliers were low, and 28% were high. Zeroes were a common outlier value, in that only one enclosure’s sample included no individuals. In addition to decreasing the number of non-normal sets, transformation of the abundances produced fewer sets with an outlier enclosure and identified more low outliers than high. These patterns are expected, given the logarithmic transformation commonly used to normalize mesocosm data (e.g., Van den Brink et al. 2000, Brock et al. 2015).

## Discussion

Aquatic mesocosm studies are regularly conducted within higher-tier risk assessment of PPPs. In this context, the individual studies are designed to capture and quantify potential effects of the chemical on complex ecosystems with numerous interacting populations and are geared to inform the specific risk assessment (Graney 1993, Caquet et al. 2000). Mesocosm studies have also been used to assess effects of other stressors such as warming, ultraviolet radiation, sedimentation, and eutrophication (Pérez et al. 2003, Yvon-Durocher et al. 2010, Fordham 2015, Ren et al. 2017, Mächler et al. 2018, Turunen et al. 2018). The interactions of taxa with their physicochemical environment and with other taxa in the food web are usually not the focus of the studies but are important drivers of population dynamics in untreated waterbodies as well as of the observed effects of stressors (Rothhaupt 2000, Fleeger 2020, Zhao et al. 2020). With the current paper, we are publishing data from untreated (control) mesocosms from 7 studies conducted at the same test facility in compliance with GLP and conducted following similar procedures, corresponding to the highest degree of standardization that can be achieved to such systems. Although the sampling design is geared towards characterizing treatment-related effects on taxa in mesocosms, the information about the dynamics of the taxon community is more comprehensive compared to the information available for natural aquatic ecosystems. To date, however, only a few studies have attempted to explore the consistency and variability in the dynamics of key parameters within aquatic mesocosms (e.g., Knauer et al. 2005). We consider the publication of the data as an opportunity for comparison to other related data sets. With the qualitative analysis of the data across studies applied in the current paper, we provide an overview of the similarities as well as variability across taxa and their dynamics in the studies. The analysis conducted is not considered exhaustive but rather provides foundation for further analyses conducted focusing on more specific questions.

Our analysis characterizes the abundance and variability of taxa across studies, setting the stage for simulations of the mesocosm systems with aquatic system models (ASMs). ASMs capture the population dynamics considering their bioenergetics, interacting physical-chemical parameters, and interactions amongst taxa in the food web. ASMs have been applied to estimate effects of PPP exposures and other stressors across taxa in multiple aquatic ecosystems (Park et al. 2008, Kattwinkel et al. 2016, Strauss et al. 2017, Bartell et al. 2018). Thereby, the models represent the ecosystem with and without exposure. The characterization of the ecosystem available from the mesocosm studies provides a unique opportunity to address whether ASMs can capture the dynamics of these systems, through calibration and validation. However, calibrating ASMs to a single data set may result in overfitting, precluding the transferability of the calibrated model to capture independent data from a different study (validation). Thus, the characterization of the abundances of taxa and their temporal dynamics needs to be addressed in the context of the variability in the empirical system (Ritter and Muñoz‐Carpena 2013, Schmolke et al. 2020). Given the variability observed across data sets in the current study, the characterization of the species composition, abundance ranges and the temporal patterns of population dynamics across studies can provide the framework in which ASMs can be expected to perform when simulating the untreated mesocosm systems.

With respect to physical and chemical parameters in aquatic mesocosms, this study documented an overall high consistency in seasonal dynamics. An outstanding example of a highly consistent pattern was documented for nitrate, one of the most bioavailable forms of nitrogen for usage of primary producers in water (Raven and Giordano 2016), which peaked in all studies during mid- to late June and sharply declined afterwards. The reason(s) for this pattern have not yet been pinpointed but might be related to denitrification, the microbial reduction of nitrate, typically occurring in water and sediment under anaerobic circumstances as well as to growth of nutrient consumers, e.g., algae and macrophytes. Other nutrients that are known to constrain growth and development of primary producers, such as phosphate (Schindler 1977, Hecky and Kilham 1988, Elser et al. 2007, Kolzau et al. 2014) did not show any identifiable consistency in patterns across the studies and/or were detected at concentrations lower or just slightly exceeding the limit of detection. Since availability of nutrients does not only influence abundance dynamics and physiology of primary producers (Ferragut and de Campos Bicudo 2012), but also affects dynamics of consumers feeding on them (Daldorph and Thomas 1991, DeMott and Van Donk 2013, Ardón et al. 2020), analytical methods with lower limits of detection might help to detect small changes in nutrient concentrations and thereby not only allowing a better understanding of the observed variability but also would provide important input to ASMs.

Primary producers, including phytoplankton, periphyton, and macrophytes, represent the basis of aquatic food webs. They affect nutrient cycling (Karpowicz et al. 2020) and influence physical parameters, such as levels of oxygen and light exposure throughout the system (Pearsall and Ullyott 1933, Tilzer et al. 1995). Gathering quantitative and qualitative information on primary producers is thus of vital importance for understanding and simulating dynamics up the food chain. The observation that within both phytoplankton and periphyton, the development of ‘green algae’, ‘blue-green algae’ and ‘diatoms’ was correlated in time, but temporally shifted, is in line with a wealth of empirical findings on competitive interactions between phytoplankton and periphyton communities, especially in terms of nutrients and light (Hansson 1988, Hansson 2006, Sánchez et al. 2010, Rodríguez and Pizarro 2015). The positive correlation (simultaneous dynamics) of the different algae groups within phytoplankton and periphyton, however, is in contrast to what is expected from the typical succession of algal communities, as they not only compete for both light and nutrients, but also exhibit varying degrees of sensitivity to grazing by different species (Steinman et al. 1987, Burson et al. 2018).

Whether the observed patterns are caused by the method used (i.e., grouping of algae based on DF excitation spectra) remains to be clarified in future studies. The composition of phytoplankton and periphyton communities exhibited pronounced fluctuations throughout the studies, with the dominant fraction in the great majority of control enclosures being green algae. While seasonal fluctuations in algae community composition can be explained by taxa-specific responses to seasonal changes in light, temperature, and nutrients (Marks and Lowe 2011), the dominance of green algae is more difficult to explain given the oligotrophic status of the enclosures, which is commonly known to favor the (co-) dominance of blue-green algae and/or diatoms (Siegfried 1985). The considerable variability that was documented in abundance and community dynamics of phytoplankton and periphyton not only across control enclosures from different years but also across those within the same years and within studies might be a result of the heterogeneous spatial distribution (patchiness) of the algae themselves as well as of herbivore taxa feeding on them, e.g., *Daphnia* (Pinel-Alloul and Ghadouani 2007). Coping with this background variability manifesting at the base of the food web could pose a major challenge for ASMs, especially when it comes to food-web based models.

Among other roles, macrophytes provide habitats, food sources, and structures for emergence and egg laying aquatic organisms (Blake 1994, Jeppesen et al. 1998). In this sense, the increase in overall macrophyte coverage that was documented over the course of all studies may not only affect light and availability of nutrients and thus the development of periphyton and phytoplankton (Søndergaard and Moss 1998, Huss and Wehr 2004), but also the abundance of other taxa which inhabit structures formed by macrophytes, feed on macrophytes directly, or on periphytic algae growing on macrophytes (Thomaz et al. 2008, Thomaz and Cunha 2010). The increase in coverage that was consistently observed among control enclosures of all studies was mainly driven by the growth of the two macrophyte taxa, *Chara globularis* and *Myriophyllum spicatum*. Both species were planted in the mesocosms according to the study design. Thus, their dominance might be highly specific to the test systems rather than indicative of a competitive superiority relative to the other macrophyte taxa. This is also in line with the fact that *Zannichellia palustris* and filamentous algae, both of which were introduced into the mesocosms with water and/or sediment taken from a natural pond, and thus by chance, occurred sporadically and were highly variable in time. Simulations by ASMs might be influenced by this variable of chance. Which taxon, i.e., *Chara globularis* or *Myriophyllum spicatum*, dominated the enclosures varied among studies, but not within studies. In this context, it is worth mentioning again that the design of mesocosm studies (including the management of the pond systems) is adapted to the particular aim of the study and initial conditions, e.g., planting density of macrophytes, vary between different studies. In addition, the variability amongst macrophyte morphology, e.g., *Zannichellia palustris* developing linear long leaves arranged on slender branching stems and *Myriophyllum spicatum* developing feather-like leaves whorled around the stems (Cook 1996), is not captured by the assessment of macrophyte coverage.

When focusing on taxa abundances, it should be considered that data represent single snapshots of complex and dynamic community structures within a limited temporal window (2-3 months). The sampling during the time window may capture the true taxa abundances, population, and community structures with varying levels of precision, dependent on the characteristics of the sampling methods used. The trapping devices used in the mesocosm studies presented in the current paper have been specifically designed to capture species potentially sensitive to chemical exposure at sufficient abundances to fulfill statistical data requirements for aquatic risk assessment. Thus, some taxa, including *Asellus aquatics* and *Cloeon* sp., both feeding on plant detritus (Bloor 2011, Banegas et al. 2020), might be overrepresented in the control data, as the supply of macrophytes and/or decomposed organic material in macroinvertebrate artificial substrate samplers (MASS) is expected to enrich these taxa relative to the surrounding sediment or water. Other taxa, in contrast, such as *Trichocerca*, a phytophilic and periphytic rotifer (Duggan 2001), and Tubificidae, sediment-dwelling oligochaete taxa (Gooderham and Tsyrlin 2002), might be severely underestimated relative to the real abundance or not detected due to their cryptic habitat and/or behavior. For other taxa, such as *Chaoborus* sp., which does not require ascending devices to emerge, the abundances recorded with the trapping devices used, could in fact be representative. In order to estimate the total abundance of each taxon and to align sampling data with species abundances simulated in ASMs, species- and sampling-specific correction factors need to be established. One approach to determine these factors would be to assess abundance *via* standard sampling methods, followed by an exhaustive characterization of entire mesocosms.

In the enclosures of all seven studies, the zooplankton communities were dominated by a few highly abundant taxa that have frequently been described as predominant within lentic European water bodies (Plaβmann et al. 1997, Antão - Geraldes and Boavida 2007, Wærvågen and Andersen 2017). These included the rotifer taxa *Keratella quadrata*, and *Polyarthra* sp. and nauplia (early larval stage of copepods). As observations on zooplankton communities in lakes, artificial ponds and water reservoirs have shown (Ramírez García et al. 2002, Rettig et al. 2006, Seebens et al. 2013, McKinstry and Campbell 2018), several zooplankton taxa exhibited pronounced intra- and inter-annual fluctuations. *Keratella quadrata*, for instance, consistently attained highest abundances in June/July and diminished vastly thereafter. Such consistent temporal pattern should be expected to be reproduced by simulations with ASMs. For multiple zooplankton taxa, however, there was no consistency evident in terms of population dynamics or total abundance, not across control enclosures of single studies nor across those of different studies. This is not surprising given that growth, survival, and reproduction, and thus abundance of zooplankton, are shaped by a complex interplay of various ‘bottom-up’ (e.g., concentration of total phosphorus, water temperature, food availability) and ‘top-down’ regulating factors (e.g., abundances of predators), not all of which are yet understood in their relative strength (Finlay et al. 2007, Ji et al. 2013, Braun et al. 2021). When ASMs are used to simulate the dynamics of those zooplankton taxa, such variability observed in the data needs to be considered. For instance, abundance ranges observed across studies can be used to define expected ranges in simulation outputs.

Among the 63 macroinvertebrate taxa found in the control enclosures were not only several taxa known to be highly sensitive to insecticides and other xenobiotics (e.g., *Chaoborus sp*. and *Cloeon dipterum*; Schroer et al. 2004, Van den Brink et al. 2016), but also key representatives from different trophic levels that spend all or part of their life cycles in freshwater bodies. These included predators (e.g., Hirudinea*)*, omnivores (e.g., Chironomidae), herbivores (e.g., *Planorbis* sp.), and detrivores (*Asellus aquaticus;* Vos et al. 2004*)*. Of the latter, additional individuals were added to the enclosures in three of the seven studies to ensure appropriate representation of crustaceans, which otherwise would have been poorly represented by presence of sparse numbers of Ostracoda and *Asellus aquaticus*. Total abundances and abundance dynamics of several macroinvertebrate taxa considerably varied among seasons, years, and studies. For emerging multivoltine taxa, including *Chaoborus* sp. and *Cloeon dipterum*, this variability might be explained by differences in physical parameters (e.g., water temperature) that not only affect timing of emergence but also length and number of generations per year (Brittain 1982, Ouimet 2008, Cockroft et al. 2019). Other factors contributing to variability in macroinvertebrate abundance and abundance dynamics could include differences in availability of food sources, macrophyte-based habitat structures, and presence or absence of overwintering generations (O’Connor 1991, Leszczyńska et al. 2017), all of which are likely to differ among ponds of different ages and thus successional stages (Hassall et al. 2012, Sferra et al. 2017). It is not surprising that the variability in total abundances of emerging insect larvae was reflected at the adult life-stages of these taxa, i.e., in their occurrence in emergence traps. As observed in zooplankton taxa, some macroinvertebrates were observed in consistently higher abundances than others across studies, allowing definition of abundance ranges by taxon (or taxon group) that should be reproduced when simulating the systems with ASMs. A few taxa, including *Chaoborus*, consistently peaked in their abundance early across studies (before mid or end of July).

Being complex and highly dynamic systems, it is reasonable to assume that variability can not only be detected between different pond systems but also between replicates within single pond systems. Although the individual mesocosms in each study were initiated with very similar starting conditions, small differences, e.g., in abundances of primary producers, or chemical parameters, are known to lead to cascading effects and distinctly divergent population and community dynamics. This effect is typically more pronounced the longer the separation lasts (Bach et al. 2016). A study comparing zooplankton and environmental variables from 12 mesocosms studies in the UK (Brooks et al. 2019) reported that the number of organisms and variation of abiotic factors can make mesocosm data exponentially complex. The authors identified a few similarities in dynamics among their studies, for example generally greater abundance of Cladocera earlier in the season. More complex series of changes in the mesocosms could involve abiotic factors, primary producers, and succession among zooplankton as the season progresses; patterns sometimes varied among replicates (Brooks et al. 2019).

The statistical comparisons of the variability of macroinvertebrate and zooplankton abundance among replicates in the seven mesocosm studies suggest that it is uncommon for one replicate to statistically deviate from the others. Among all sets, each of which is defined as one taxon within one study on one date, 92% had no enclosures different enough from the mean to trigger statistical significance. This number is worth to be stressed out again, considering the complexity of the test systems and the long duration of the studies. Moreover, the enclosures that were significantly different should not necessarily be interpreted as incorrect or outliers; instead, they provided unique information, or described a unique setting, not observed in the remaining enclosures. Differences are expected, but large differences were uncommon overall. This analysis method did not distinguish sets in which two enclosures may be deviant, nor sets in which more enclosures’ measurements were widely spaced. These tests were run on data transformed in a way commonly used in analysis of treatment effects in mesocosms; this helped normalize the data, but also revealed that zeroes (enclosures with no taxon presence) sometimes served as a downward skew. Overall, based on this metric, sampling and setup methods used within each study frequently created relatively similar conditions among replicate enclosures, and may have captured similar representations of populations of organisms from one enclosure to the next. This is an important prerequisite for the assessment of treatment effects from an exposure to a pesticide (or other stressors) and for the simulation of the mesocosm ecosystem with ASMs.

Although we could show a single replicate did not generally deviate from the others within the same study, variability among enclosures within a study was observed. In addition, considerable variability was observed between studies. Such variability in systems following similar study designs can be attributed to differences weather conditions and methodological errors and points to the amplification of small differences in initial conditions over time as well as stochastic processes. Accordingly, ASMs cannot be expected to predict the system dynamics to a high accuracy despite their consideration of complex interactions in the food web. However, by simulating ‘top-down’ and ‘bottom-up’ regulating factors and mechanisms, ASMs might help to characterize the relative strengths of the regulating factors and their interplay. In addition, ASMs can be used to identify gaps in our understanding of the systems and inform data collection in aquatic mesocosms that help filling these gaps. Starting with publication of data from mesocosm studies obtained under the highest possible degree of standardization, provides a sound database that opens up new opportunities for development of ASMs. We are aware that the analysis conducted in this study is only one of many ways the data could be analyzed and thus not the end of the story - hence we welcome re-analysis of the data under further aspects.

Now, several challenges remain to be overcome allowing for the alignment between data collected from the mesocosms and simulations of these systems with ASMs. First, the main objective of mesocosm studies is the characterization of effects from specific stressors. Accordingly, the experimental and sampling design is optimized to detect potentially sensitive species and other case-specific endpoints. ASMs are developed to simulate aquatic ecosystems in their entirety, focusing on biomass flows through the whole food web. Thus, the biomass of key taxonomic groups in the mesocosm needs to be established to better inform ASMs, this is still rarely done given this is not requirement in mesocosm studies. Secondly, taxonomic and food web composition in ASMs need to reflect the food web composition in mesocosm studies, which may be study specific. Third, interactions between abiotic and biotic compartments, as well as all relevant trophic interactions need to be identified. Finally, for a relevant comparison between observed and simulated mesocosm dynamics, sources of uncertainty and data gaps in the empirical data have to be characterized.

## Conclusion

Mesocosm studies are conducted to capture effects of stressors to aquatic ecosystems, but do not aim to characterize the complex ecosystem itself. In this analysis, we present data from untreated mesocosms from seven studies, providing an insight into the species present in these artificial ecosystems and the consistency and variability in their dynamics over time. While single replicates (enclosures) did not deviate statistically within a study, considerably variability across mesocosm studies emphasizes the complexity of interactions that drive the dynamics in these systems. ASMs are designed to capture this complexity and could be used to simulate observed dynamics and predict dynamics under untested conditions. To be able to compare simulated systems with empirical data, it is important to consider uncertainties in the data and the comparability of simulated and empirical community composition that are informed by similar measures. Future research should focus on existing knowledge gaps, e.g., by implementing additional sampling methodologies to mesocosm studies that allow for better understanding of the test system as a whole or even allow for conversion of standard mesocosm data to allow for comparison with model outputs (e.g., conversion of abundance data into biomass data). Gaps to be addressed by implementation of additional sampling methodologies include biomass of algae, macrophytes, macroinvertebrates and zooplankton organisms by length, (bio-) volume and weight measures; taxa-specific sampling efficiencies and thus representativeness of the samples for the test system as a whole (accessible *via* exhaustive whole mesocosm samplings), dynamics of nutrients by using analytical methods with lower detection limits and global radiation through measurements on-site. The better understanding of the underlying dynamics of ecosystems can lead to a better understanding of the complex interactions with stressors. This understanding can inform the use of ASMs as tools in risk assessment.

## Supplementary information

Supplementary Dataset S1

Supplementary Dataset S2

Supplementary Dataset S3

Supplementary Dataset S4

Supplementary Dataset S5

Supplementary Dataset S6

Supplementary Dataset S7

Supplementary Material

Supplementary Dataset S8
