# Supplementary material for: Characterization of patterns and variability in the dynamics of outdoor aquatic mesocosms: exploring the capabilities and challenges in data supporting aquatic system models
Source: Ecotoxicology. 2023 Jul 25;32(6):782–801. doi: 10.1007/s10646-023-02685-0 (PMC10449964; doi:10.1007/s10646-023-02685-0)
Supplement: Supplementary file 8 — Supplementary Material [file 10646_2023_2685_MOESM8_ESM.docx]

**Supplementary Information**

**Characterization of patterns and variability in the dynamics of aquatic mesocosms: exploring the capabilities and challenges in data supporting aquatic system models**

**Author names and affiliations:**

Ann-Kathrin Loerracher ^A^_,_ Jürgen Schmidt ^A^, Peter Ebke ^A^,^,^ Amelie Schmolke ^B^, Farah Abi-Akar ^B,^ Nika Galic ^C^, Roman Ashauer ^C,D^

A: Mesocosm GmbH, Homberg (Ohm), Hesse, Germany

B: Waterborne Environmental, Leesburg, Virginia, USA

C: Syngenta Crop Protection AG, Basel, Switzerland

D: Department of Environment and Geography, University of York, UK

**E-mail address of the corresponding author:** annkathrin.loerracher@mesocosm.de

**SI Table 1:** Study-specific details on the introduction of organisms

| **Name of data set**  **(Mesocosm GmbH ID)** | **Species** | **Date of introduction** | **Individuals**  **per**  **enclosure** | **Origin** |
| --- | --- | --- | --- | --- |
| Control 16a  (1478-10) | *Trichoptera* | 13/06/2016 | 15 | Natural water body |
|  | *Gammarus pulex* | 13/06/2016 | 25 |  |
|  | *Gammarus roeseli* | 13/06/2016 | 25 |  |
|  | *Asellus aquaticus* | 16/06/2016 | 25 | Ponds on test facility |
| Control 18a  (1404-48) | *Asellus aquaticus* | 25/04/2018 | 30 | Ponds on test facility |
|  |  | 02/05/2018 | 15 |  |
|  |  | 11/05/2018 | 15 |  |
|  | *Gammarus pulex* | 19/04/2018 | 50 | Natural water body |
|  |  | 08/05/2018 | 20 |  |
| Control 18c  (1502-02) | *Gammarus pulex* | 08/05/2018 | 50 | Natural water body |
|  |  | 11/05/2018 | 30 |  |
| Control 19a  (1510-02) | *Asellus aquaticus* | 17/05/2019 | 50 | Ponds on test facility |
|  | *Gammarus pulex* | 21/05/2019 | 20 |  |
| Control 19b  (1404-50) | *Lymnaea stagnalis* | 17/05/2019 | 4 | Ponds on test facility |
|  | *Planorbis planorbis* | 17/05/2019 | 4 |  |
|  | *Radix* sp. | 17/05/2019 | 2 |  |

**SI Table 2:** Study-specific details on in-life management actions

| **Name of data set**  **(Mesocosm GmbH ID)** | **Type of management action** | **Date** |
| --- | --- | --- |
| Control 18b  (1506-02) | Installation of a tent – reduction of the photosynthetic activity to mitigate the rise in pH associated with photosynthesis. | 25/05/2018 – 07/06/2018 |
| Control 18c  (1502-02) | Installation of a tent – reduction of the photosynthetic activity to mitigate the rise in pH associated with photosynthesis. | 25/05/2018 – 07/06/2018 |
|  | Aeration of all enclosures | 02/08/2018 – 25/08/2018 |
| Control 19a  (1510-02) | Installation of pumps – generation of a slight vertical circulation. The pumps were switched off at least 15 minutes prior to each sampling event and were switched on again after the sampling was completed. | 21/05/2019 – 16/08/2019 |

**SI Table 3:** Study specific details on the number of emergence traps and enclosure area covered by emergence traps [%].

| Name of data set  (Mesocosm GmbH ID) | Number of emergence traps per enclosure | Enclosure area covered by emergence traps [%] |
| --- | --- | --- |
| Control 14a  (1478-04) | 1 | 7.8 |
| Control 16a  (1478-10) | 2 | 15.6 |
| Control 18a  (1404-48) | 2 | 15.6 |
| Control 18b  (1506-02) | 1 | 22.3 |
| Control 18c  (1502-02) | 2 | 18.9 |
| Control 19a  (1510-02) | 2 | 15.6 |
| Control 19b  (1404-50) | 1 | 22.3 |

**SI Table 4:** Detail of the transformations used for the comparison of replicates, based on Van den Brink et al., 2000.

|  | **Macroinvertebrates** | **Zooplankton** |
| --- | --- | --- |
| Smallest non-zero measurement (x_min_) | 1 individual | 0.06 individuals/L |
| Calculation to derive coefficient from Ax_min_ = 2 | A(1) = 2 | A(0.06) = 2 |
| Coefficient A used in transformations | A = 2 | A = 33.33 |
| Transformation for all measurements | ln(2x + 1) | ln(33.33x + 1) |

**SI Table 5:** Correlation coefficients of chlorophyll-a measured in blue-green and diatom algae in periphyton and phytoplankton by study and enclosure (correlations shown in SI Figure 10, panel A).

| **Name of data set** | **Enclosure** | **Periphyton (ug chl-a/area)** | **Phytoplankton (ug chl-a/L)** |
| --- | --- | --- | --- |
| **Control 14a** | 1 | 0.58 | -0.54 |
| **Control 14a** | 2 | 0.83 | 0.12 |
| **Control 14a** | 3 | 0.87 | 0.37 |
| **Control 14a** | 4 | 0.39 | 0.56 |
| **Control 14a** | 5 | 0.09 | 0.26 |
| **Control 14a** | 6 | 0.56 | 0.61 |
| **Control 16a** | 1 | 0.11 | 0.9 |
| **Control 16a** | 2 | 0.78 | 0.95 |
| **Control 16a** | 3 | 0.42 | 0.9 |
| **Control 16a** | 4 | 0.3 | 0.89 |
| **Control 16a** | 5 | 0.27 | 0.93 |
| **Control 16a** | 6 | 0.39 | 0.81 |
| **Control 18a** | 1 | 0.9 | 0.41 |
| **Control 18a** | 2 | 0.98 | 0.45 |
| **Control 18a** | 3 | 0.99 | 0.22 |
| **Control 18a** | 4 | 0.91 | 0.44 |
| **Control 18a** | 5 | 0.53 | 0.26 |
| **Control 18b** | 1 | 0.99 | 0.75 |
| **Control 18b** | 2 | 0.85 | 0.31 |
| **Control 18b** | 3 | 0.02 | 0.83 |
| **Control 18b** | 4 | 0.89 | 0.72 |
| **Control 18b** | 5 | 0.98 | 0.5 |
| **Control 18c** | 1 | 0.55 | 0.6 |
| **Control 18c** | 2 | 0.26 | 0.8 |
| **Control 18c** | 3 | 0.89 | 0.99 |
| **Control 18c** | 4 | 0.03 | 0.93 |
| **Control 18c** | 5 | -0.08 | 0.83 |
| **Control 19a** | 1 | 0.54 | 0.56 |
| **Control 19a** | 2 | 0.72 | 0.41 |
| **Control 19a** | 3 | 0.89 | 0.59 |
| **Control 19a** | 4 | 0.89 | -0.06 |
| **Control 19a** | 5 | 0.97 | 0.26 |
| **Control 19b** | 1 | 0.71 | 0.78 |
| **Control 19b** | 2 | 0.55 | 0.83 |
| **Control 19b** | 3 | -0.38 | 0.97 |
| **Control 19b** | 4 | 0.41 | 0.94 |
| **Control 19b** | 5 | 0.83 | 0.88 |
| **Control 19b** | 6 | 0.54 | 0.74 |

**SI Table 6:** Correlation coefficients of chlorophyll-a measured in blue-green and green algae in periphyton and phytoplankton by study and enclosure (correlations shown in SI Figure 10, panel B).

| **Name of data set** | **Enclosure** | **Periphyton (ug chl-a/area)** | **Phytoplankton (ug chl-a/L)** |
| --- | --- | --- | --- |
| **Control 14a** | 1 | 0.9 | -0.29 |
| **Control 14a** | 2 | 0.76 | 0.45 |
| **Control 14a** | 3 | 0.94 | 0.01 |
| **Control 14a** | 4 | 0.8 | -0.06 |
| **Control 14a** | 5 | 0.78 | 0.96 |
| **Control 14a** | 6 | 0.93 | 0.3 |
| **Control 16a** | 1 | 0.15 | 0.5 |
| **Control 16a** | 2 | 0.86 | 0.7 |
| **Control 16a** | 3 | 0.93 | 0.96 |
| **Control 16a** | 4 | 0.05 | 0.76 |
| **Control 16a** | 5 | 0.25 | 0.88 |
| **Control 16a** | 6 | 0.63 | 0.83 |
| **Control 18a** | 1 | 0.63 | 0.36 |
| **Control 18a** | 2 | 0.86 | -0.2 |
| **Control 18a** | 3 | 0.93 | -0.35 |
| **Control 18a** | 4 | 0.99 | 0.15 |
| **Control 18a** | 5 | 0.67 | 0.39 |
| **Control 18b** | 1 | 0.93 | -0.34 |
| **Control 18b** | 2 | 0.98 | 0.03 |
| **Control 18b** | 3 | 0.16 | 0.5 |
| **Control 18b** | 4 | 0.37 | 0.69 |
| **Control 18b** | 5 | 0.98 | 0.03 |
| **Control 18c** | 1 | 0.94 | 0.18 |
| **Control 18c** | 2 | 0.56 | 0.68 |
| **Control 18c** | 3 | 0.94 | 0.95 |
| **Control 18c** | 4 | 0.41 | 0.98 |
| **Control 18c** | 5 | 0.69 | 0.87 |
| **Control 19a** | 1 | 0.72 | 0.51 |
| **Control 19a** | 2 | 0.88 | 0.43 |
| **Control 19a** | 3 | 0.97 | 0.09 |
| **Control 19a** | 4 | 0.98 | -0.54 |
| **Control 19a** | 5 | 0.98 | -0.17 |
| **Control 19b** | 1 | 0.17 | 0.6 |
| **Control 19b** | 2 | 0.59 | 0.4 |
| **Control 19b** | 3 | 0.19 | 0.81 |
| **Control 19b** | 4 | 0.5 | 0.49 |
| **Control 19b** | 5 | 0.08 | -0.35 |
| **Control 19b** | 6 | 0.69 | -0.3 |

**SI Table 7:** Correlation coefficients of chlorophyll-a measured in diatom and green algae in periphyton and phytoplankton by study and enclosure (correlations shown in SI Figure 10, panel C).

| **Name of data set** | **Enclosure** | **Periphyton (ug chl-a/area)** | **Phytoplankton (ug chl-a/L)** |
| --- | --- | --- | --- |
| **Control 14a** | 1 | 0.78 | 0.74 |
| **Control 14a** | 2 | 0.74 | 0.51 |
| **Control 14a** | 3 | 0.97 | 0.63 |
| **Control 14a** | 4 | 0.64 | 0.27 |
| **Control 14a** | 5 | 0.59 | 0.4 |
| **Control 14a** | 6 | 0.72 | 0.74 |
| **Control 16a** | 1 | 0.89 | 0.67 |
| **Control 16a** | 2 | 0.8 | 0.76 |
| **Control 16a** | 3 | 0.61 | 0.92 |
| **Control 16a** | 4 | 0.68 | 0.57 |
| **Control 16a** | 5 | 0.58 | 0.78 |
| **Control 16a** | 6 | 0.94 | 0.64 |
| **Control 18a** | 1 | 0.84 | 0.49 |
| **Control 18a** | 2 | 0.86 | -0.02 |
| **Control 18a** | 3 | 0.96 | 0.16 |
| **Control 18a** | 4 | 0.94 | -0.03 |
| **Control 18a** | 5 | 0.85 | 0.7 |
| **Control 18b** | 1 | 0.97 | 0.06 |
| **Control 18b** | 2 | 0.9 | 0.69 |
| **Control 18b** | 3 | 0.5 | 0.8 |
| **Control 18b** | 4 | 0.43 | 0.7 |
| **Control 18b** | 5 | 1 | 0.29 |
| **Control 18c** | 1 | 0.78 | 0.49 |
| **Control 18c** | 2 | 0.92 | 0.86 |
| **Control 18c** | 3 | 0.93 | 0.95 |
| **Control 18c** | 4 | 0.78 | 0.97 |
| **Control 18c** | 5 | 0.61 | 0.92 |
| **Control 19a** | 1 | 0.49 | 0.17 |
| **Control 19a** | 2 | 0.95 | 0.63 |
| **Control 19a** | 3 | 0.97 | 0.59 |
| **Control 19a** | 4 | 0.96 | 0.7 |
| **Control 19a** | 5 | 0.99 | -0.06 |
| **Control 19b** | 1 | 0.63 | 0.87 |
| **Control 19b** | 2 | 0.9 | 0.76 |
| **Control 19b** | 3 | 0.78 | 0.73 |
| **Control 19b** | 4 | 0.93 | 0.59 |
| **Control 19b** | 5 | 0.56 | -0.08 |
| **Control 19b** | 6 | 0.94 | -0.02 |


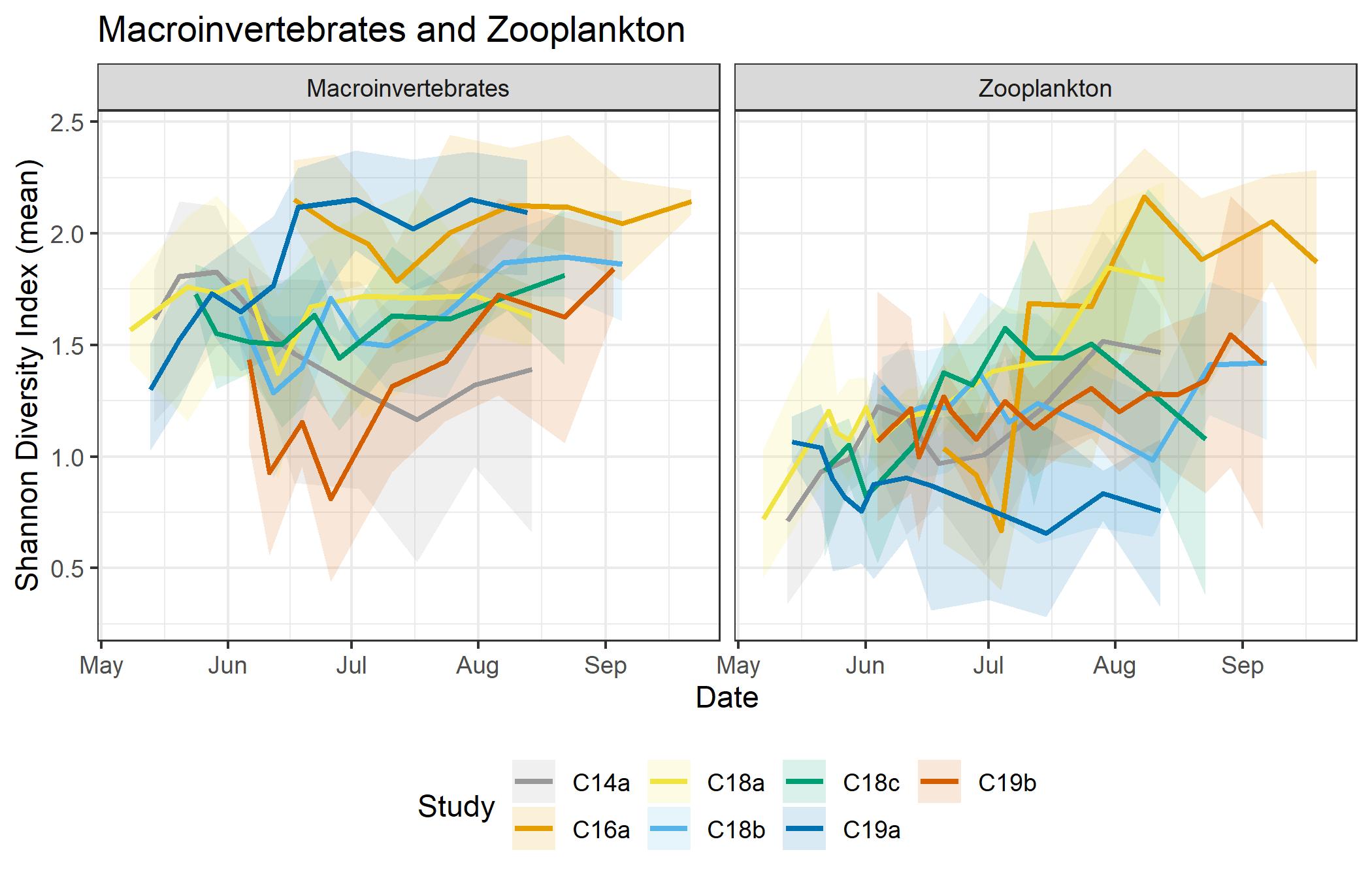
**SI Figure 1:** Shannon Diversity Index across time. Lines are means of enclosures with shading spanning the minimum to maximum values.

**
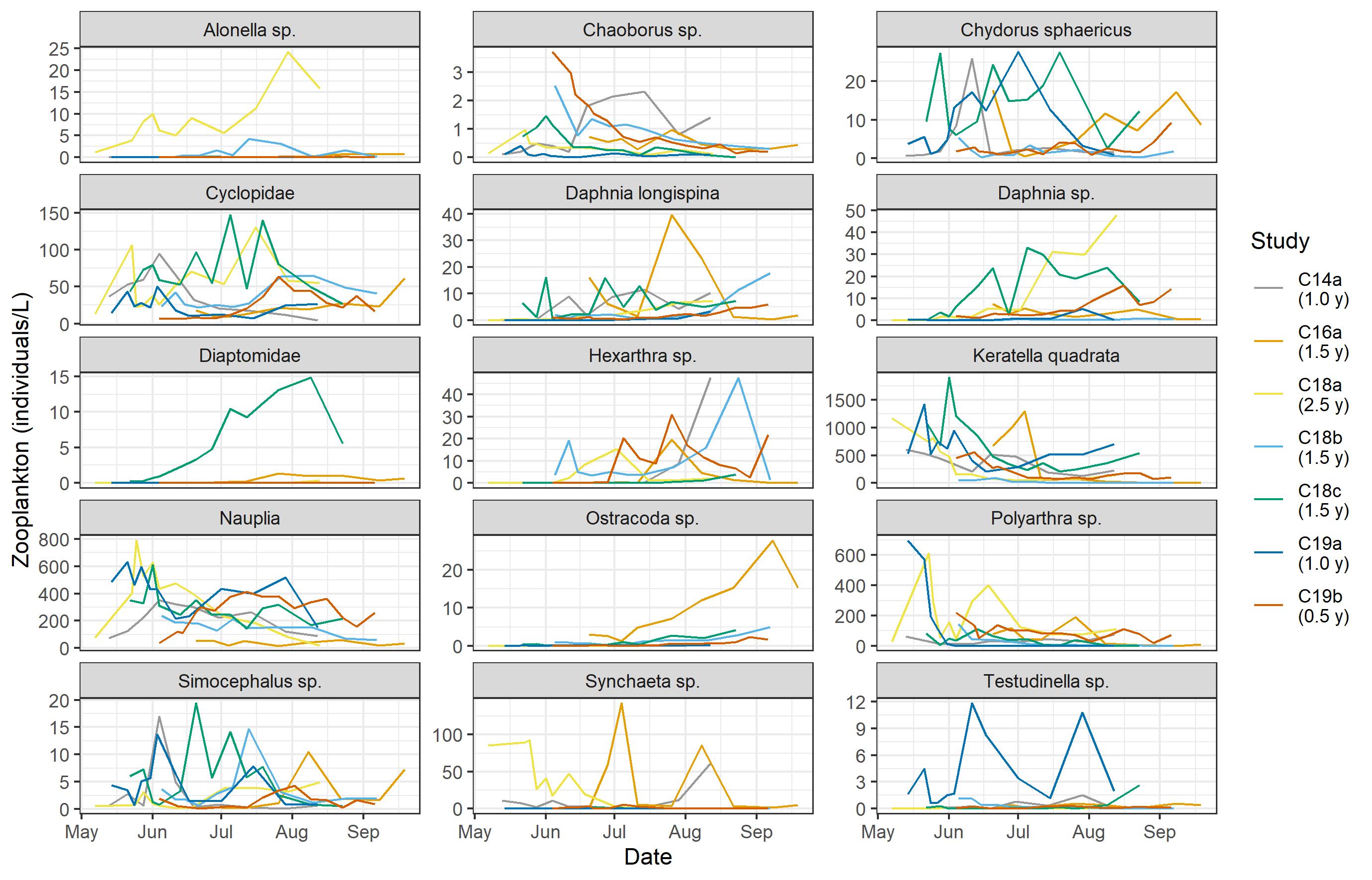
**

**SI Figure. 2:** Time lines of the zooplankton taxa that had at least 90% presence in at least one study. Lines are coloured by study. Each line depicts the mean of the enclosures within studies. Starting pond system age in years is listed after study names.

**
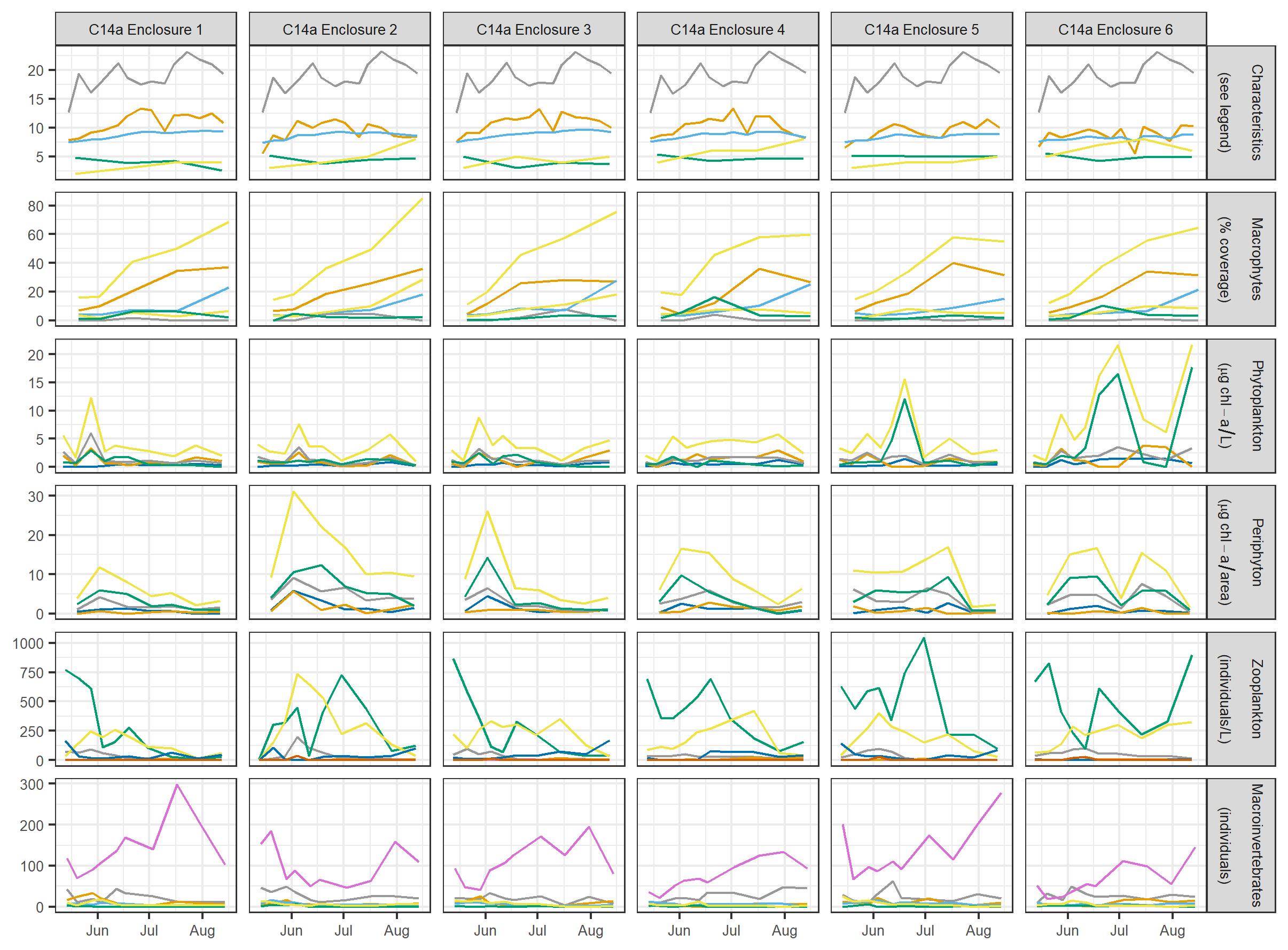

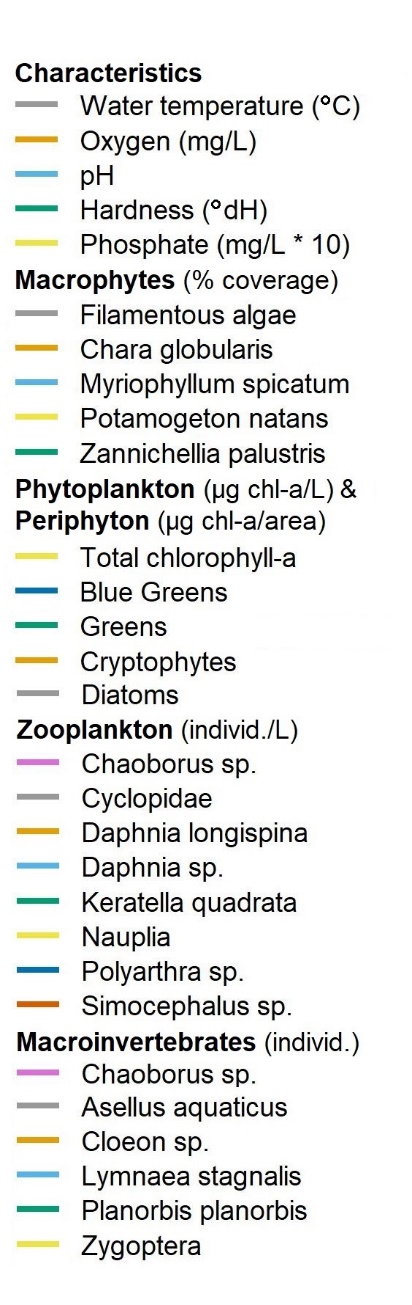
**

**SI Figure 3:** For study C14a, time lines of measured characteristics, plants, and animals (labelled in rows of graphs) are graphed by enclosure (numbered as columns of graphs). Y-axis units are specified in the legend.

**
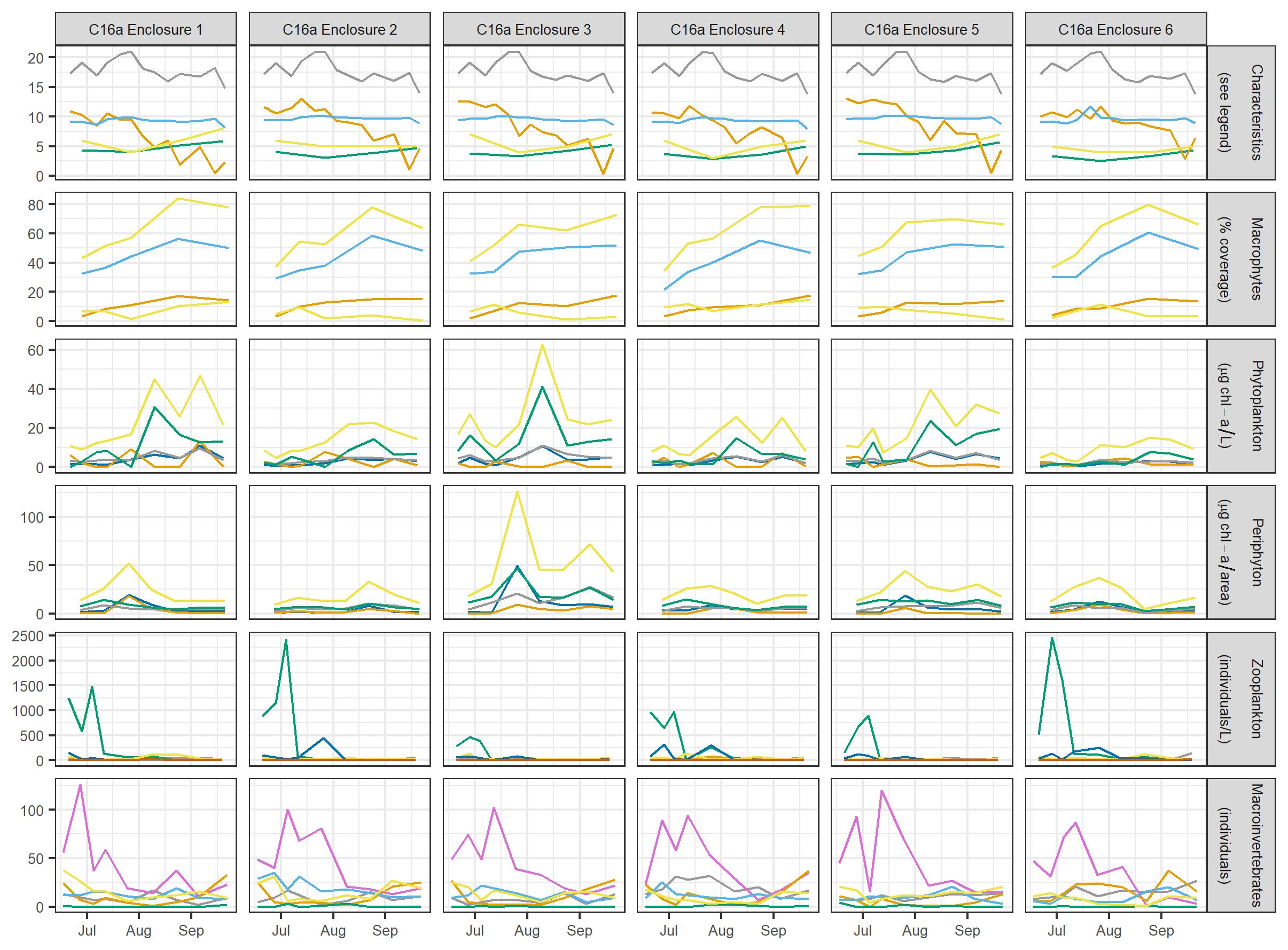

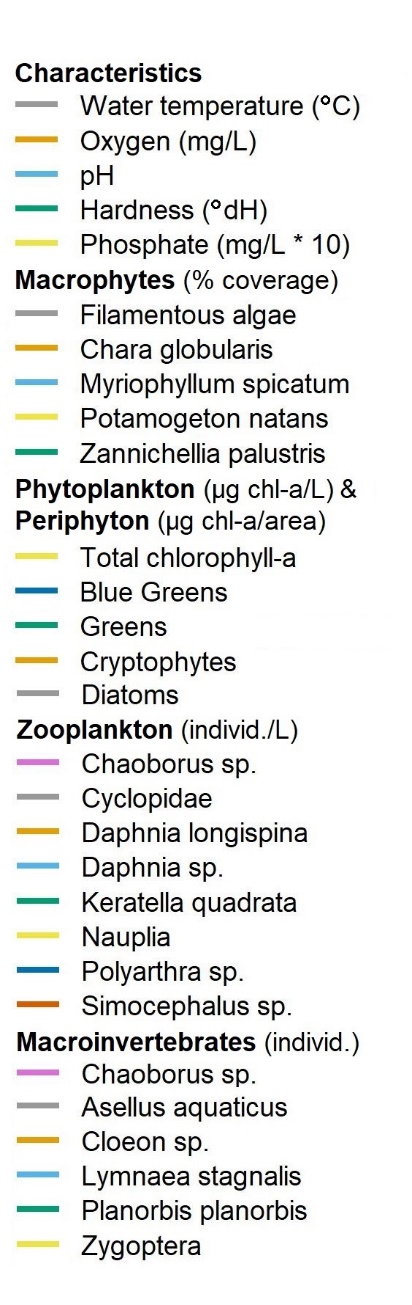
**

**SI Figure 4:** For study C16a, time lines of measured characteristics, plants, and animals (labelled in rows of graphs) are graphed by enclosure (numbered as columns of graphs). Y-axis units are specified in the legend.

**
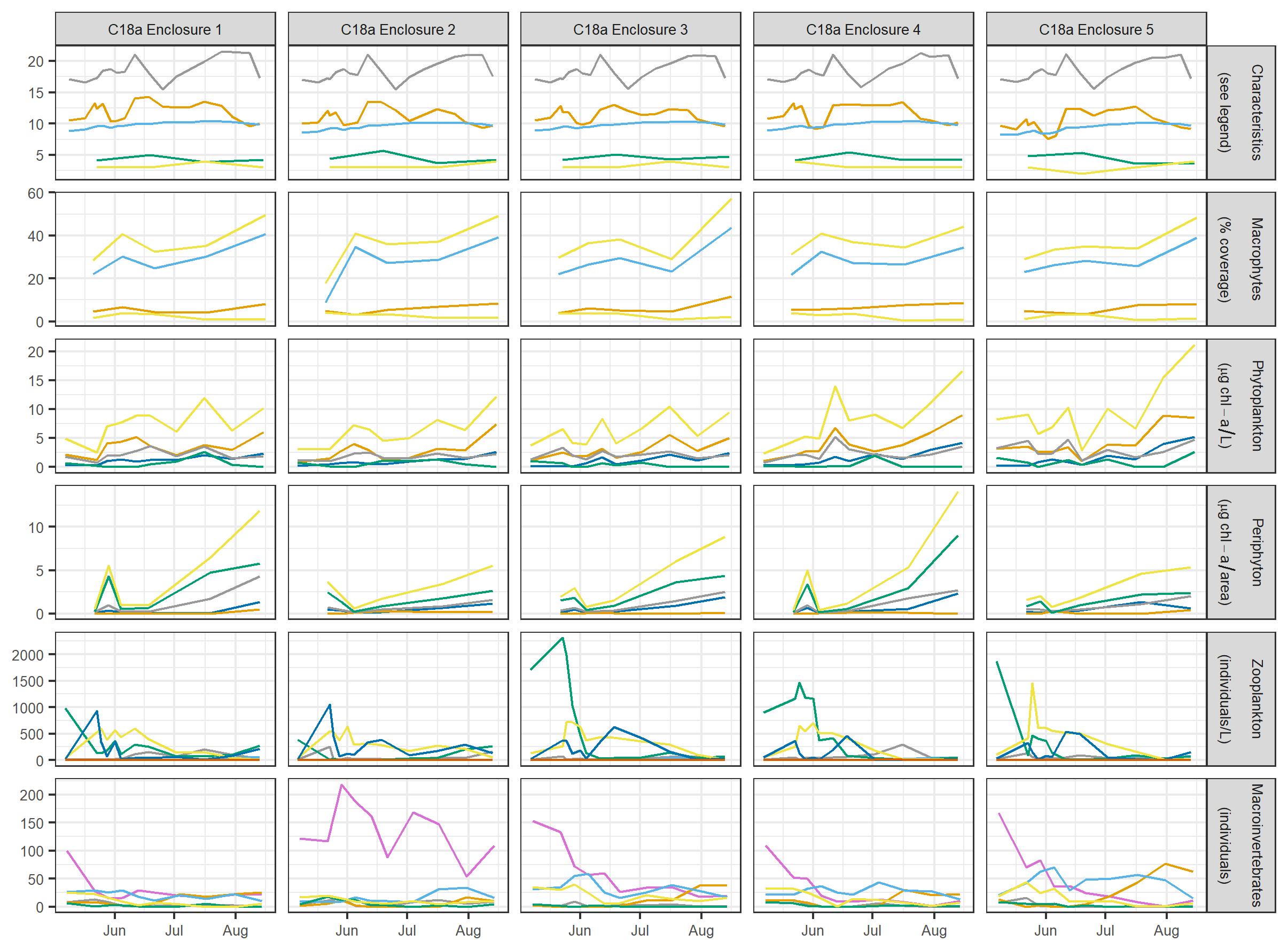

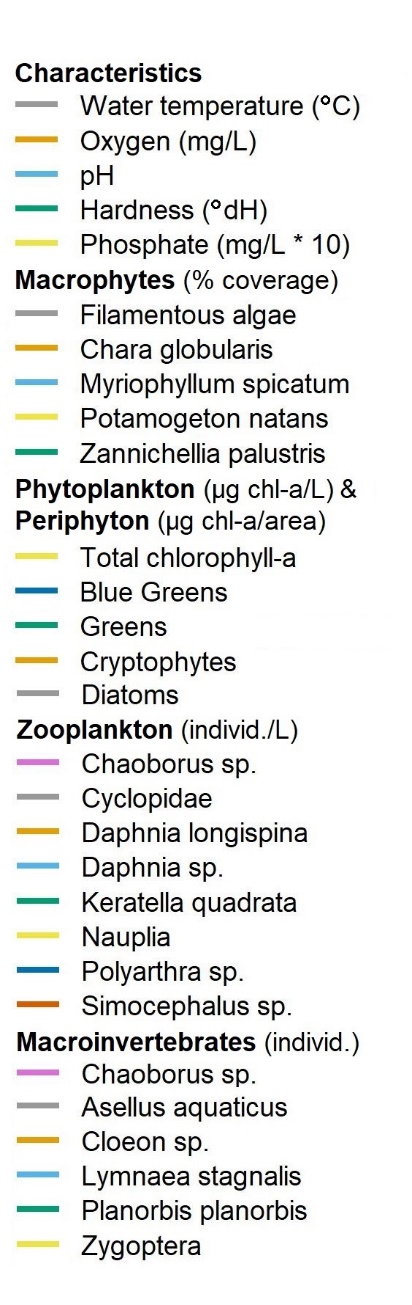
**

**SI Figure 5:** For study C18a, time lines of measured characteristics, plants, and animals (labelled in rows of graphs) are graphed by enclosure (numbered as columns of graphs). Y-axis units are specified in the legend.

**
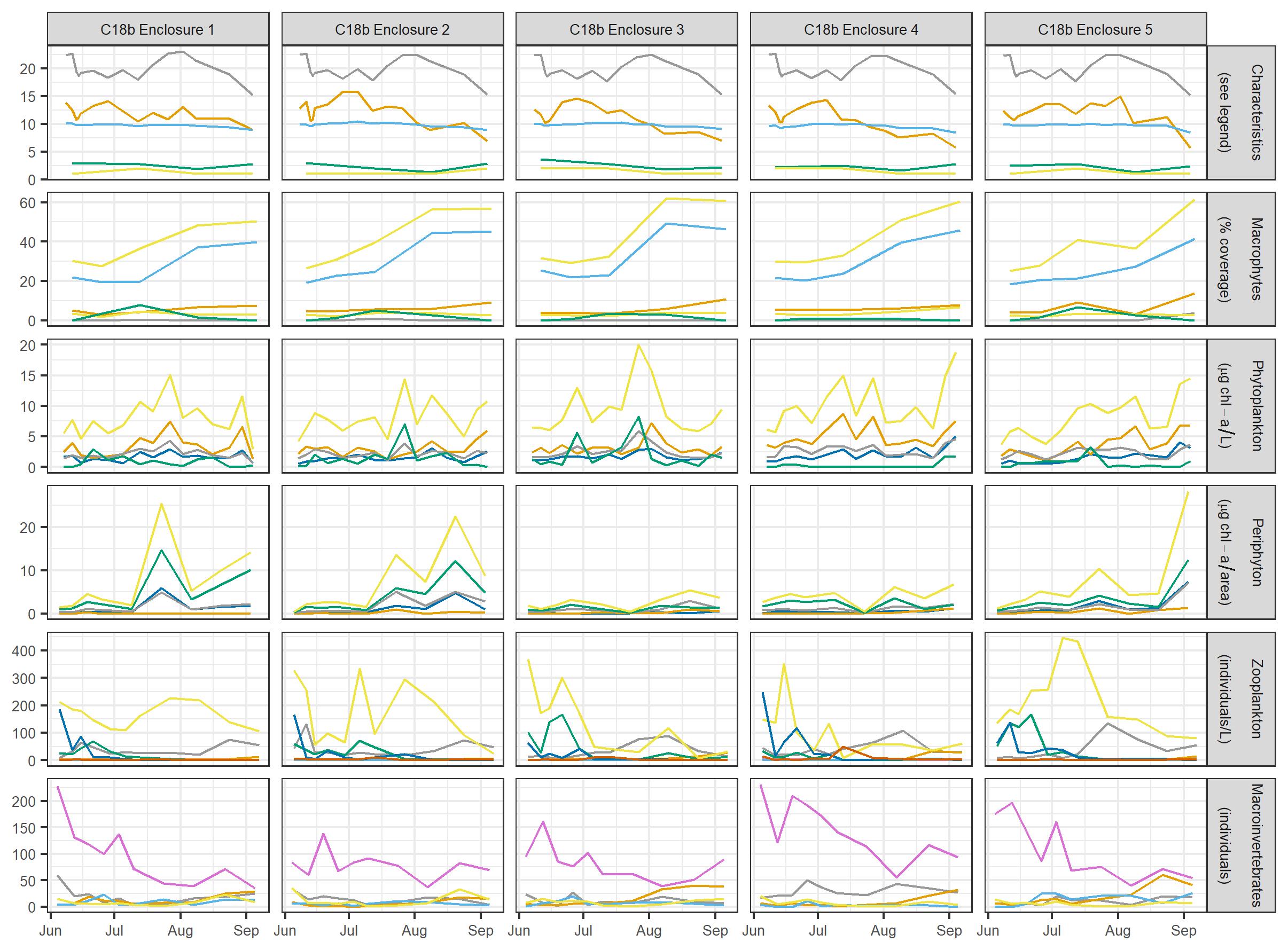

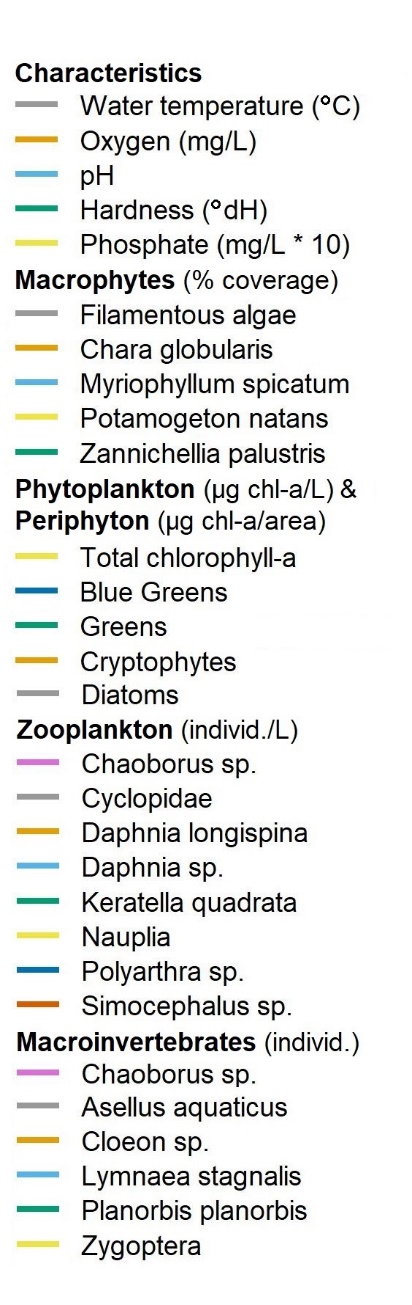
**

**SI Figure 6:** For study C18b, time lines of measured characteristics, plants, and animals (labelled in rows of graphs) are graphed by enclosure (numbered as columns of graphs). Y-axis units are specified in the legend.

**
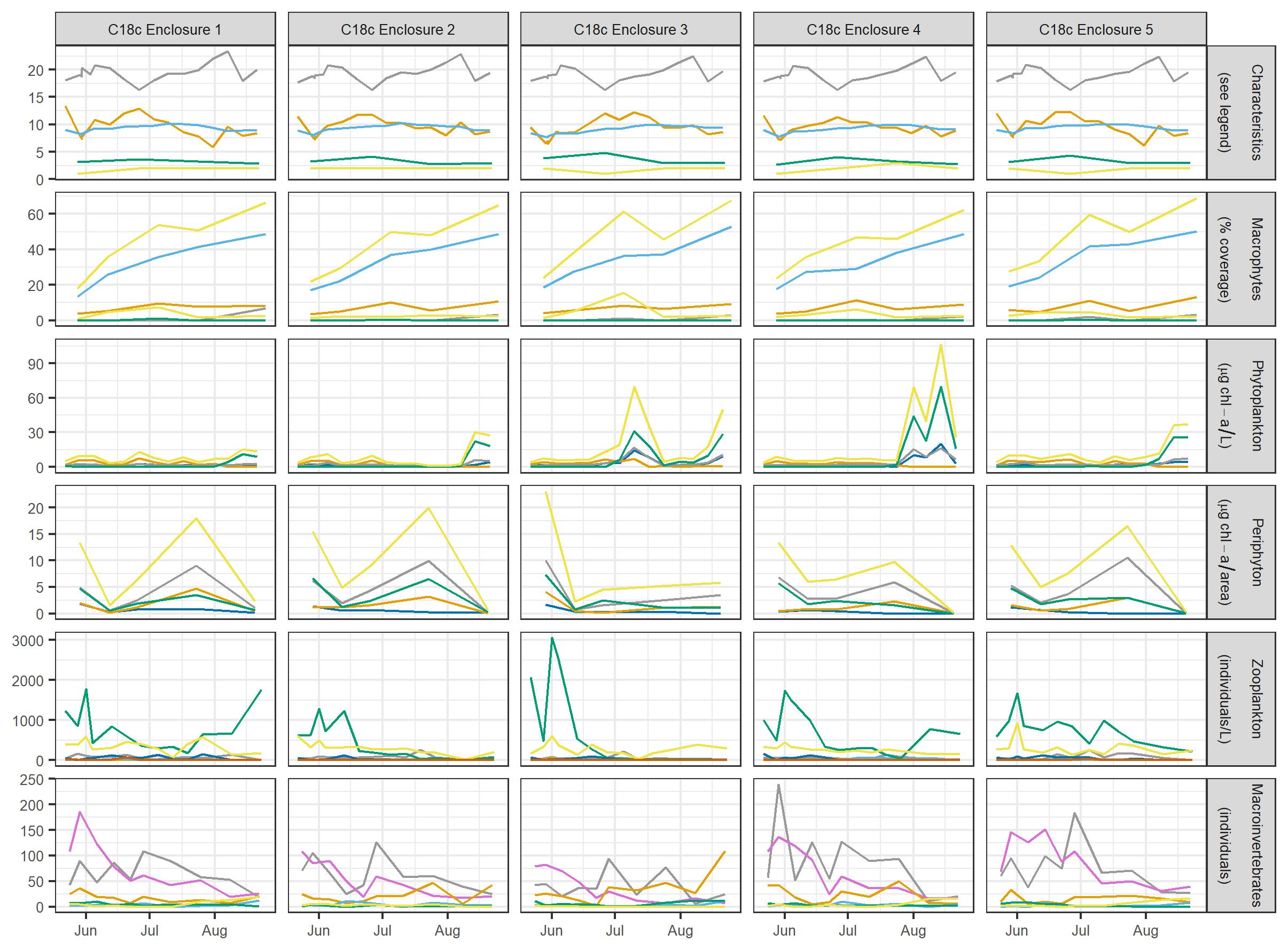

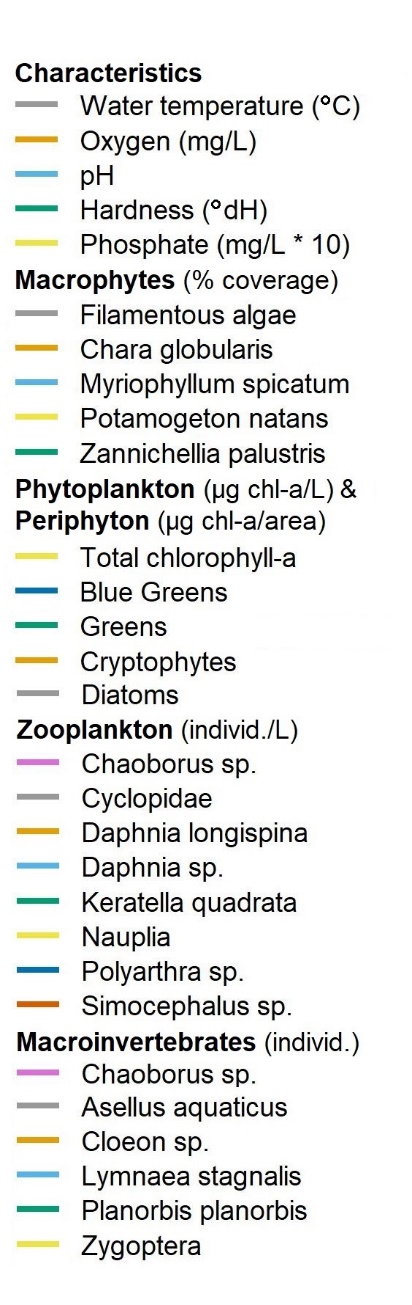
**

**SI Figure 7:** For study C18c, time lines of measured characteristics, plants, and animals (labelled in rows of graphs) are graphed by enclosure (numbered as columns of graphs). Y-axis units are specified in the legend.

**
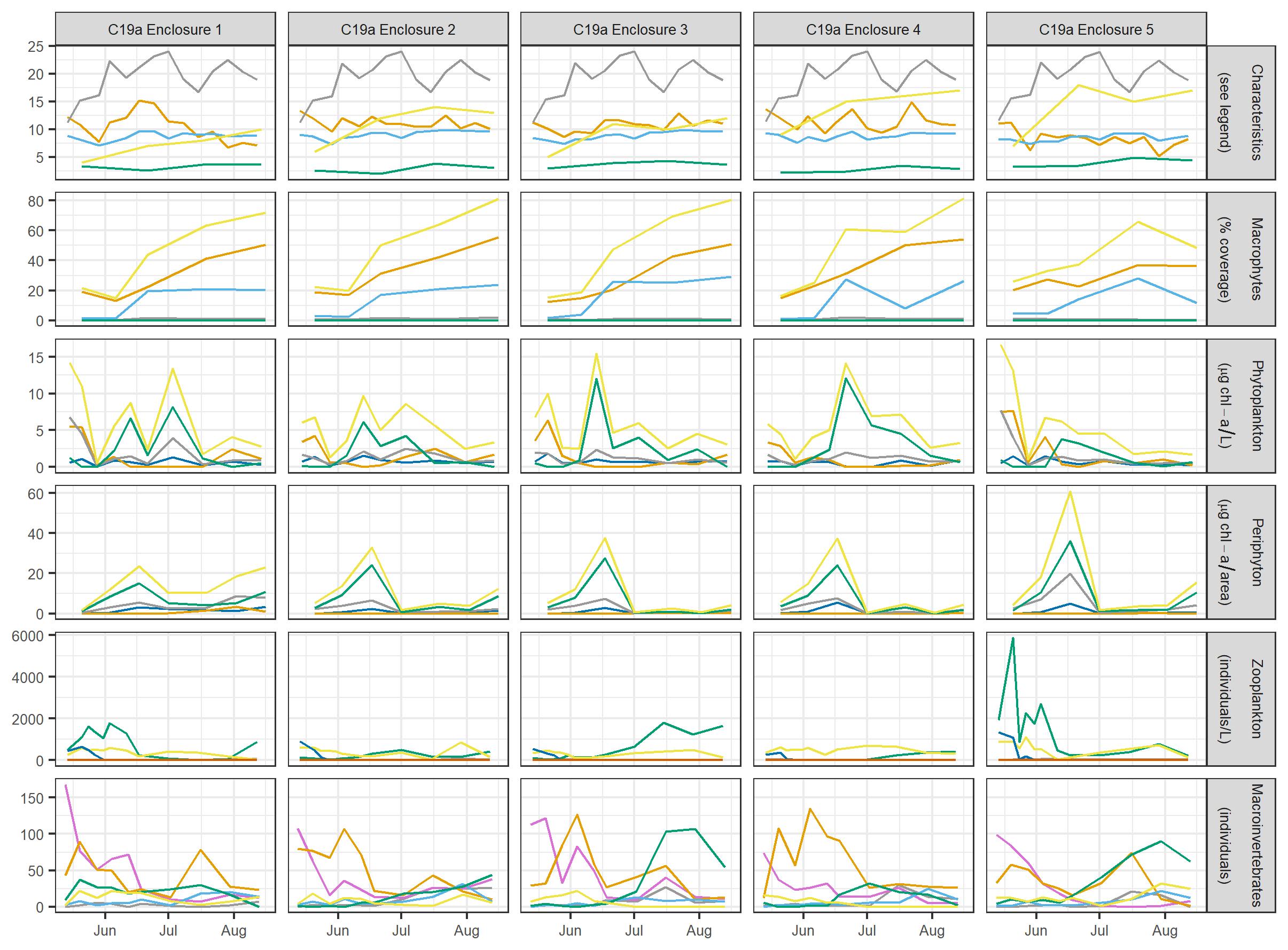

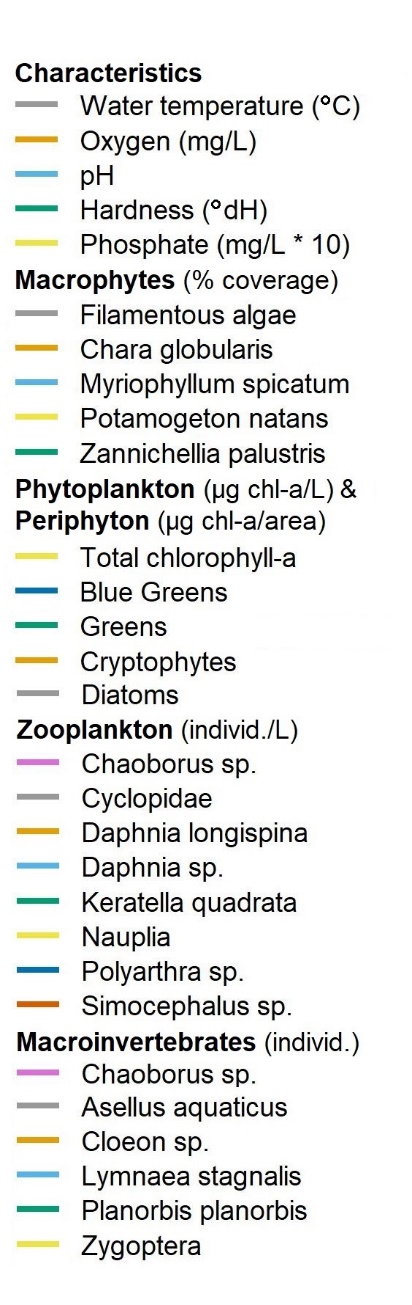
SI Figure 8:** For study C19a, time lines of measured characteristics, plants, and animals (labelled in rows of graphs) are graphed by enclosure (numbered as columns of graphs). Y-axis units are specified in the legend.

**
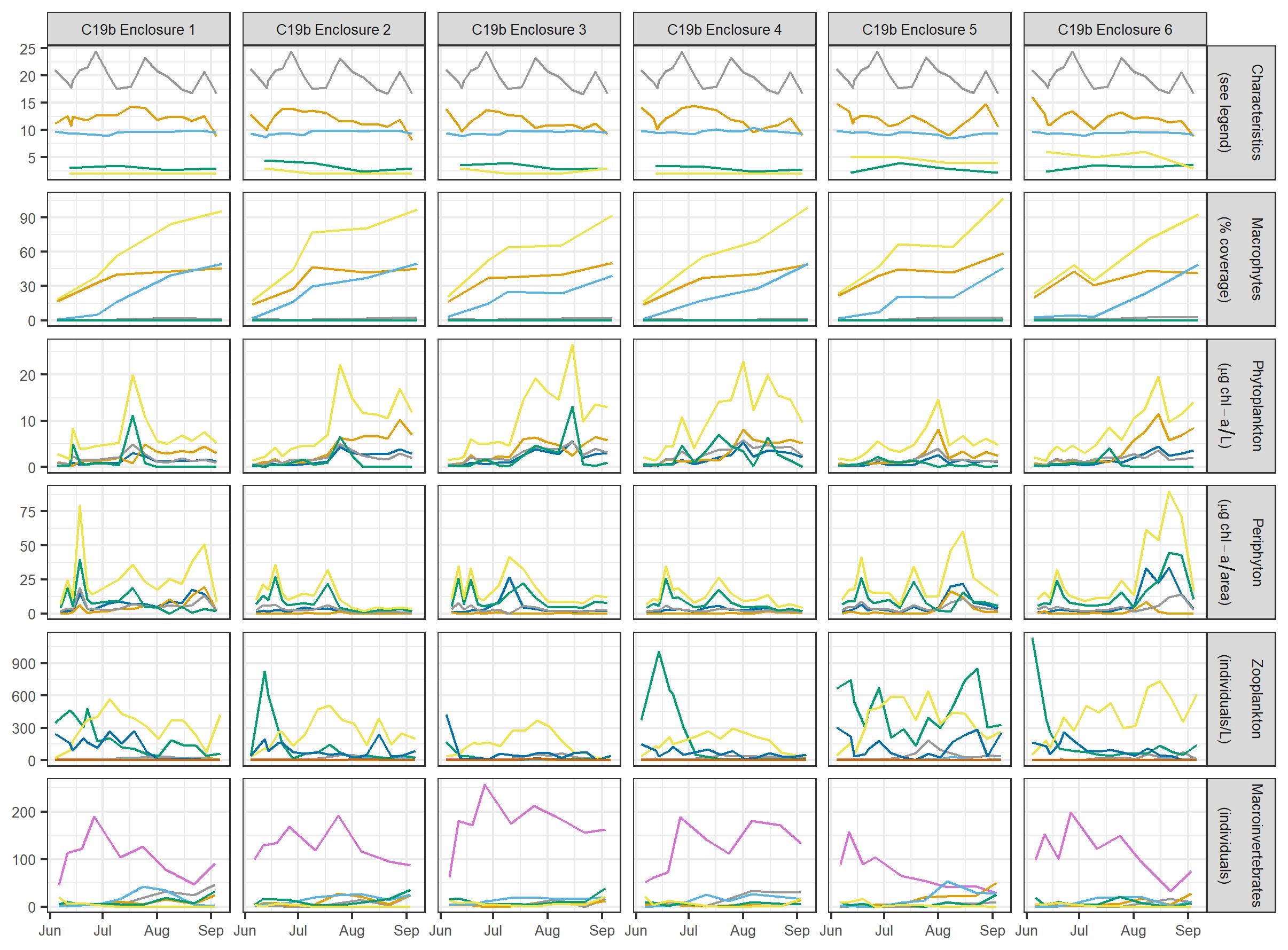

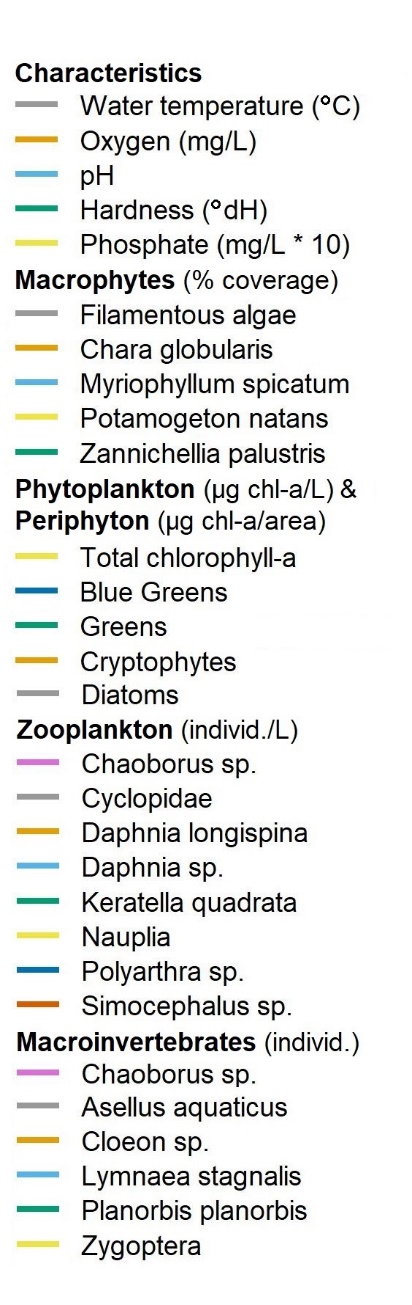
**

**SI Figure 9:** For study C19b, time lines of measured characteristics, plants, and animals (labelled in rows of graphs) are graphed by enclosure (numbered as columns of graphs). Y-axis units are specified in the legend.


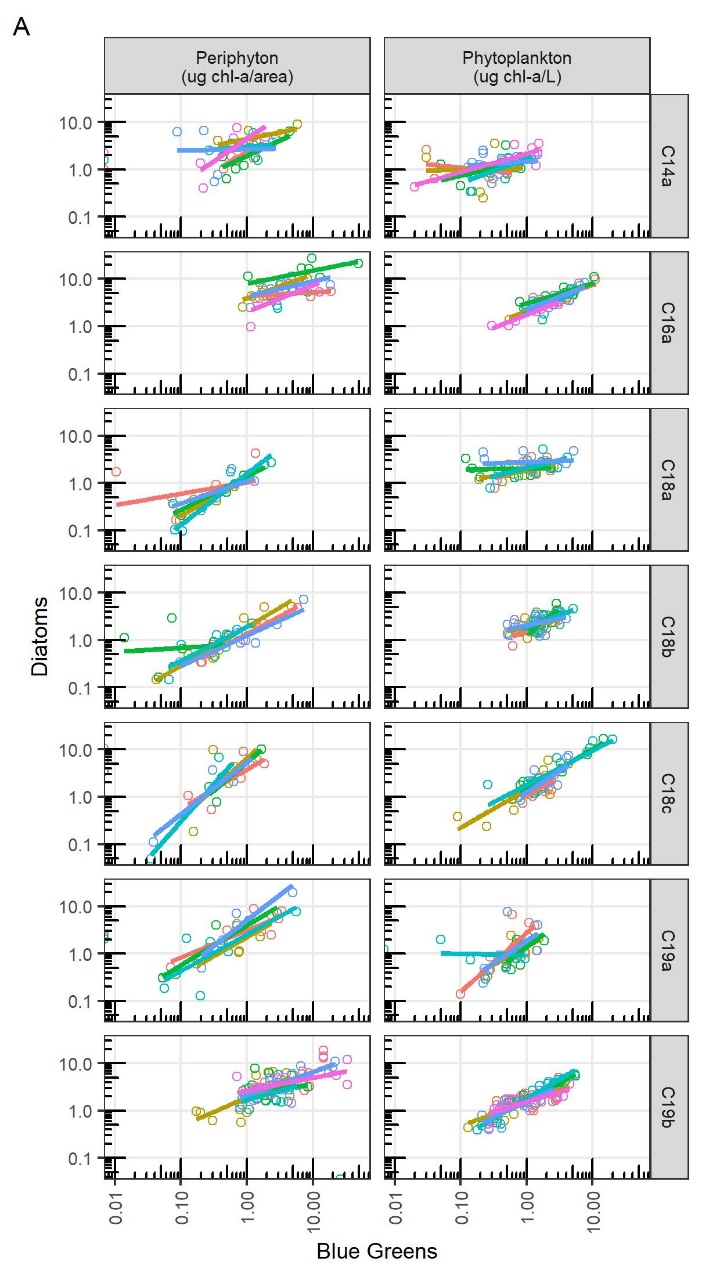

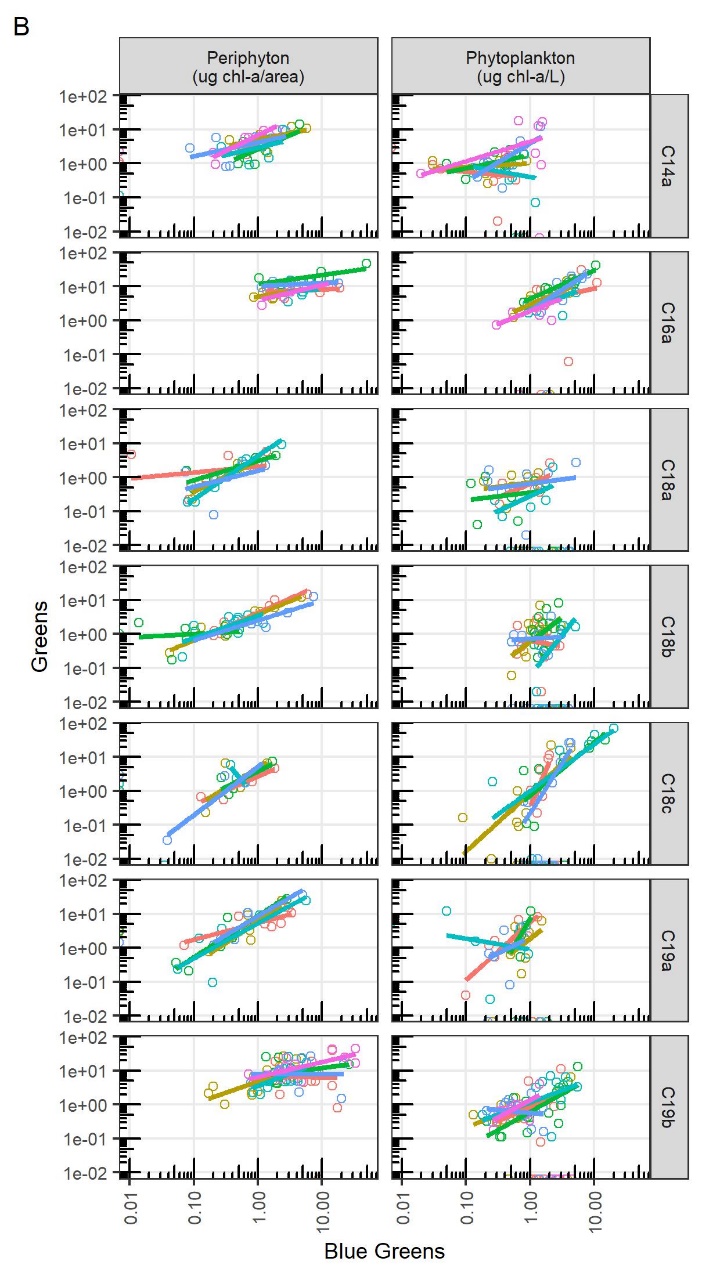

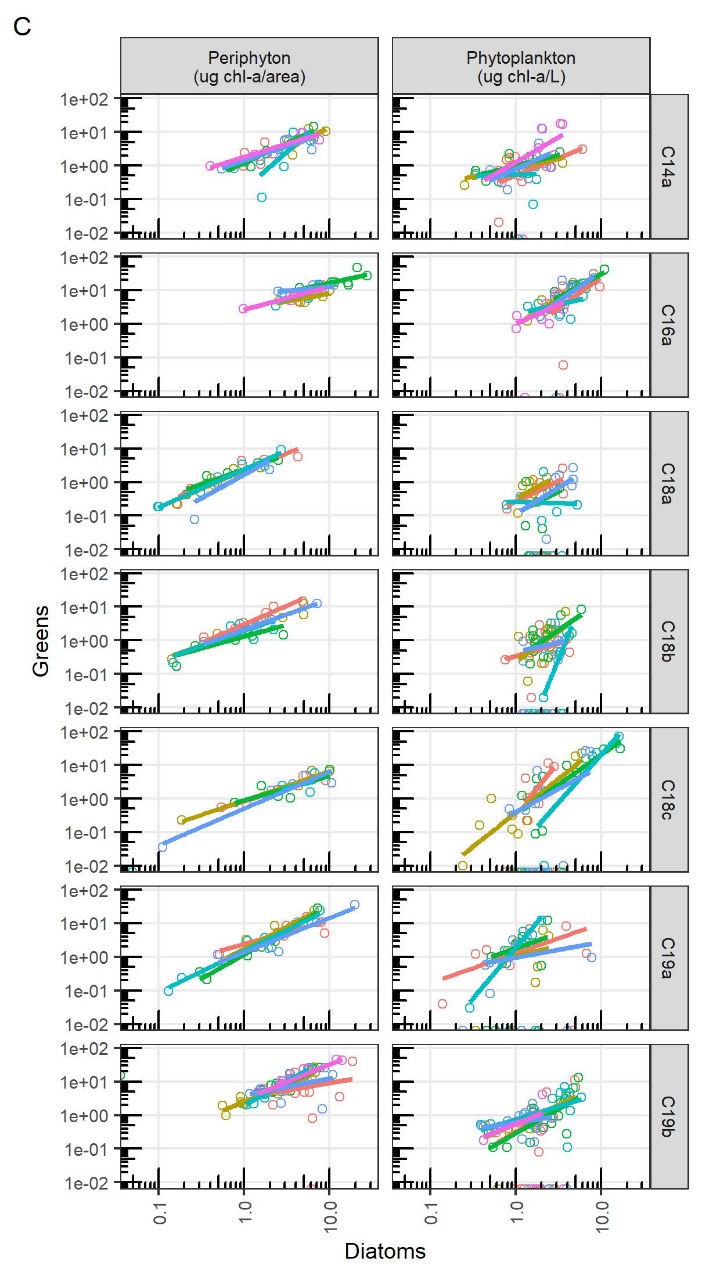


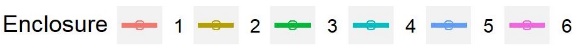


**SI Figure 10**: Correlations among chlorophyll-*a* measured in periphyton and phytoplankton A) diatoms vs. blue greens, B) greens vs. blue greens and C) greens vs. diatoms. Individual graphs are labelled by study.


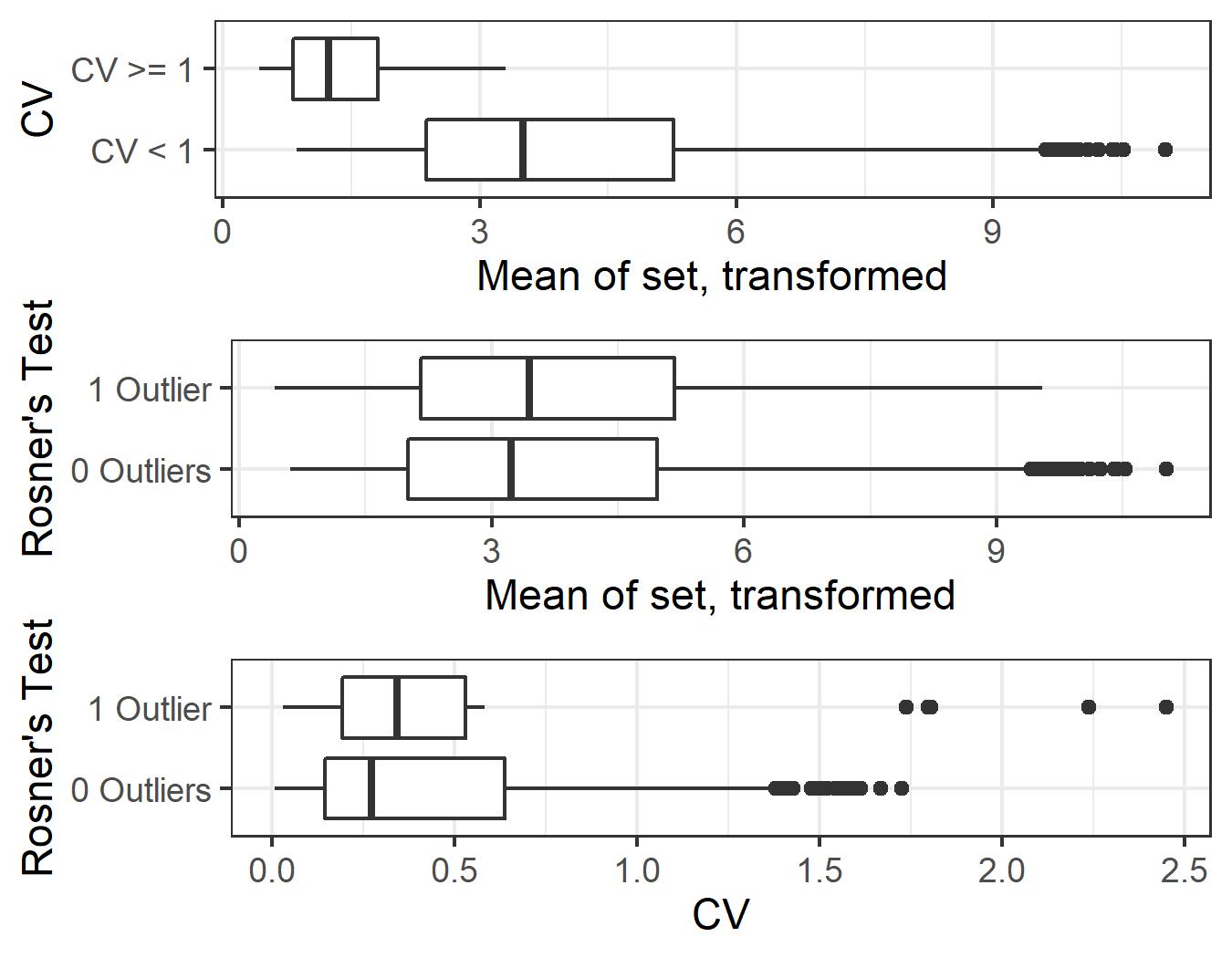


**SI Figure 11**: Box plots comparing ranges and results among CVs, Rosner’s test, and means (shown in transformed units). Top: CVs were very influenced by means, such that sets that did not have small means could not produce a CV greater than 1, regardless of their variability. Middle: Rosner’s test was not similarly affected, as it identified sets with outlier enclosures across mean values. Bottom: sets with a Rosner’s test outlier mostly had low CVs (<0.6), demonstrating that except for several extreme values, CVs did not successfully identify sets with a deviant enclosure.


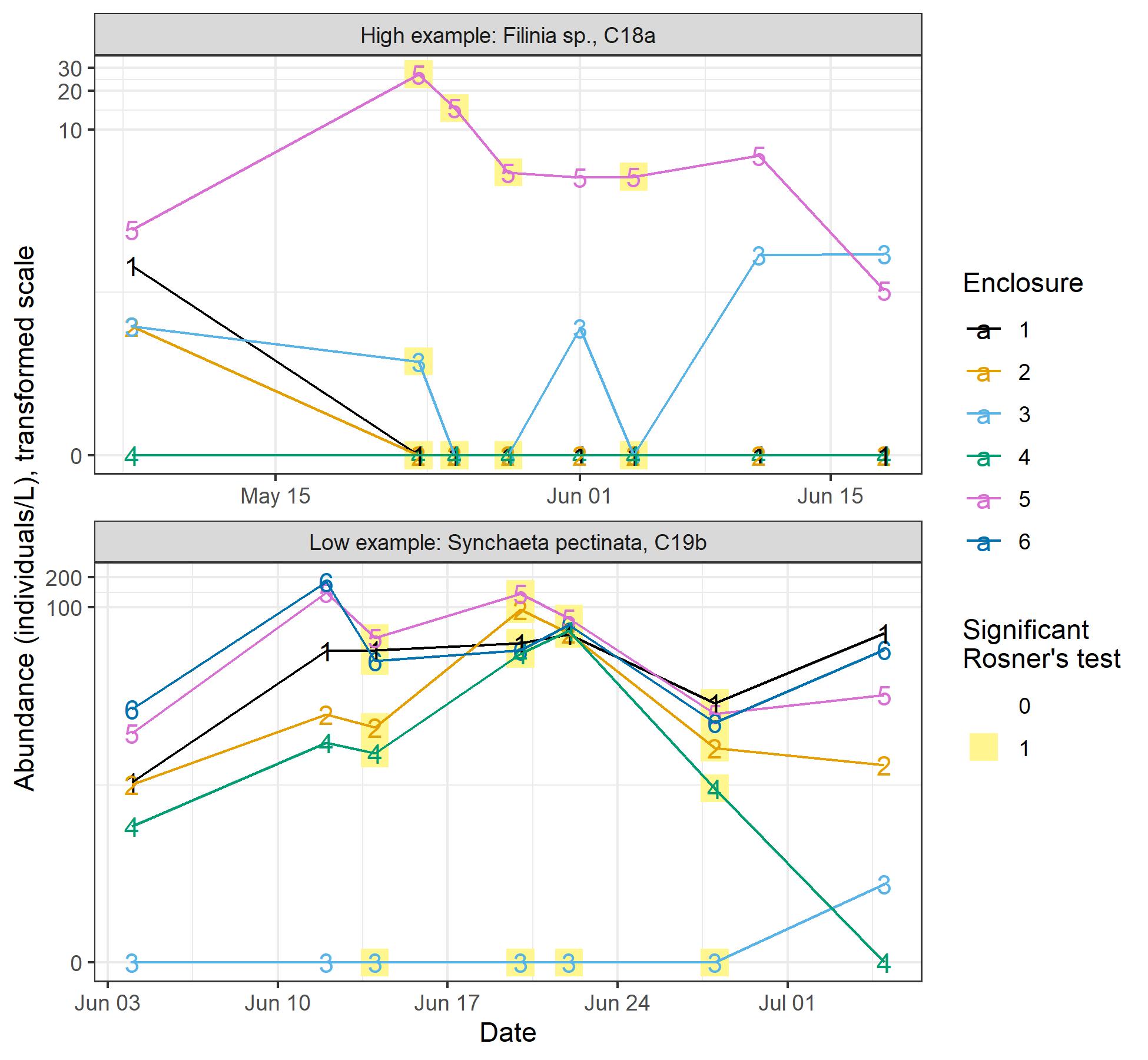


**SI Figure 12**: Examples of taxa and studies in which one enclosure was high on four dates (top plot) or low on four dates (bottom plot). Dates identified as having a significant outlier by Rosner’s test are highlighted in yellow, evaluated within a vertical set of points. The Y axis is on the ln(33.33x+1) scale, as zooplankton abundances were transformed prior to testing. Only these two taxa in these studies had up to four date sets flagged by Rosner’s test; all the remainder had fewer.
